# Oas1b-dependent Immune Transcriptional Profiles of West Nile Virus Infection in the Collaborative Cross

**DOI:** 10.1534/g3.117.041624

**Published:** 2017-06-05

**Authors:** Richard Green, Courtney Wilkins, Sunil Thomas, Aimee Sekine, Duncan M. Hendrick, Kathleen Voss, Renee C. Ireton, Michael Mooney, Jennifer T. Go, Gabrielle Choonoo, Sophia Jeng, Fernando Pardo-Manuel de Villena, Martin T. Ferris, Shannon McWeeney, Michael Gale

**Affiliations:** * Department of Immunology, University of Washington, Seattle, Washington 98109; † Center for Innate Immunity and Immune Disease (CIIID), University of Washington, Seattle, Washington 98109; ‡ OHSU Knight Cancer Institute, Oregon Health & Science University, Portland, Oregon; § Division of Bioinformatics and Computational Biology, Department of Medical Informatics and Clinical Epidemiology, Oregon Health & Science University, Portland, Oregon; ** Oregon Clinical and Translational Research Institute, Oregon Health & Science University, Portland, Oregon 97239; †† Department of Genetics, University of North Carolina at Chapel Hill, North Carolina; ‡‡ Lineberger Comprehensive Cancer Center, University of North Carolina at Chapel Hill, North Carolina 27514

**Keywords:** Oas, flavivirus, viral infection, innate immunity, multiparental populations, Multi-parent Advanced Generation Inter-Cross (MAGIC), MPP

## Abstract

The oligoadenylate-synthetase (*Oas*) gene locus provides innate immune resistance to virus infection. In mouse models, variation in the *Oas1b* gene influences host susceptibility to flavivirus infection. However, the impact of *Oas* variation on overall innate immune programming and global gene expression among tissues and in different genetic backgrounds has not been defined. We examined how *Oas1b* acts in spleen and brain tissue to limit West Nile virus (WNV) susceptibility and disease across a range of genetic backgrounds. The laboratory founder strains of the mouse Collaborative Cross (CC) (A/J, C57BL/6J, 129S1/SvImJ, NOD/ShiLtJ, and NZO/HlLtJ) all encode a truncated, defective *Oas1b*, whereas the three wild-derived inbred founder strains (CAST/EiJ, PWK/PhJ, and WSB/EiJ) encode a full-length OAS1B protein. We assessed disease profiles and transcriptional signatures of F1 hybrids derived from these founder strains. F1 hybrids included wild-type *Oas1b* (F/F), homozygous null *Oas1b* (N/N), and heterozygous offspring of both parental combinations (F/N and N/F). These mice were challenged with WNV, and brain and spleen samples were harvested for global gene expression analysis. We found that the *Oas1b* haplotype played a role in WNV susceptibility and disease metrics, but the presence of a functional *Oas1b* allele in heterozygous offspring did not absolutely predict protection against disease. Our results indicate that *Oas1b* status as wild-type or truncated, and overall *Oas1b* gene dosage, link with novel innate immune gene signatures that impact specific biological pathways for the control of flavivirus infection and immunity through both *Oas1b*-dependent and independent processes.

WNV is a mosquito transmitted flavivirus that emerged from Africa and is now endemic within Asia, middle Eastern Europe, Australia, and the Americas (Courtney *et al.* 2012). WNV is an enveloped virus carrying a genome of a positive sense, single-stranded RNA of roughly 11,000 nucleotides in length. While only 20% of individuals infected with WNV develop symptoms (www.cdc.gov/westnile), symptomatic individuals can develop clinical illness ranging from West Nile fever to encephalitis or meningitis linked with severe inflammation in the brain and spinal cord, and leading to death (Graham *et al.* 2016). WNV is among a group of emerging flaviviruses including dengue virus, Zika Virus, and Japanese encephalitis virus that are global health concerns. Understanding their molecular pathogenesis is a major step toward a therapeutic or adjunctive therapy.

During viral infection, the infected cell senses viral replication products, including viral nucleic acid, as foreign, nonself macromolecules through the actions of pathogen recognition receptors (PRRs), including RIG-I-like receptors (RLRs), Toll-like receptors (TLRs), and other PRRs (Suthar *et al.* 2013). In particular, flaviviruses including WNV are sensed by the RLRs through binding of viral RNA (Loo and Gale 2008, 2011; Errett *et al.* 2013). RNA binding induces RLR activation and interaction with downstream signaling proteins that activate transcription factors IRF3, IRF7, and NF-κB to drive innate immune gene expression and the production of type 1 and III interferons (IFNs). IFN is secreted by the infected cell to signal through the IFN receptors on both the infected cell and on bystander cells, to drive the expression of hundreds of IFN-stimulated genes (ISGs) across the local tissue (Loo and Gale 2011). Innate immune genes and ISGs have antiviral and immune modulatory activity to limit viral replication and spread such that their induction and function are essential for the control of WNV infection and immunity (Daffis *et al.* 2009; Suthar *et al.* 2010; Lazear *et al.* 2013; Lazear and Diamond 2015).

The *Oas* genes including *Oas1b* are ISGs whose expression is induced by IFN in most cell types (Elkhateeb *et al.* 2016, Choi *et al.* 2015). In humans, the *Oas* family contains *Oas1*, *Oas2*, *Oas3*, and Oas-like (*OasL*) genes. *Oas2* and *Oas3* genes have high human–mouse sequence similarity with a 1:1 orthogonal copy. The mouse *Oas* gene cluster is located on chromosome 5 and includes *Oas1*, *Oas2*, *Oas3*, and *OasL* genes. The *Oas1* gene has eight orthogonal copies (*Oas1a*, *Oas1b*, *Oas1c*, *Oas1d*, *Oas1e*, *Oas1f*, *Oas1g*, and *Oas1h*) in mouse compared to one copy (*Oas1*) in humans on chromosome 12. These eight mouse *Oas1* genes are the result of gene duplications, rearrangements, and other evolutionary processes [Choi *et al.* 2015, for more details of such genome regions in the CC, see Morgan *et al.* (2017)]. Most members of the *Oas* gene family encode 2′-5′ oligoadenylate (2-5A) enzymatic activity that catalyzes adenosine into 2′-5′-linked oligonucleotides referred to as oligoadenylates or 2-5A (Kristiansen *et al.* 2011). 2-5A serves as a ligand to bind and activate ribonuclease L (RNaseL), a latent endoribonuclease. When activated during viral infection, RNaseL serves to suppress viral replication by nucleolytic targeting viral and host RNAs. RNaseL products of RNA degradation can also serve as activator ligands of the RLRs, thus stimulating and amplifying further rounds of innate immune signaling (Malathi *et al.* 2007, Siddiqui *et al.* 2012, Drappier *et al.* 2015). Among the *Oas* family members, *Oas1b* lacks 2-5A activity, revealing that the antiviral function of *Oas1b* is unique and independent of RNaseL activation for controlling WNV infection (Scherbik *et al.* 2006; Courtney *et al.* 2012; Elbahesh *et al.* 2011). Oas1b is found truncated in classical inbred mouse strains due the presence of a stop codon/nonsense mutation within the mRNA (Elbahesh *et al.* 2011). When otherwise expressed as a full-length protein, OAS1b provides functional antiviral activity against WNV (Elbahesh *et al.* 2011). Recent studies found that OAS1b has a C-terminus domain targeting the endoplasmic reticulum with binding partner protein ABCF3 assisting in antiviral activity (Courtney *et al.* 2012). The same study showed that full-length (nontruncated) *Oas1b* inhibited OAS1a activity and lowered 2-5A levels in a dose-dependent manner *in vivo*. Polymorphisms in *Oas1b* are also thought to impact the overall host immune response to virus infection, but how *Oas1b* operates in immune regulation is not defined (Bigham *et al.* 2011).

*In vivo* studies assessing *Oas1b* function during WNV infection and immunity have been largely conducted with traditional inbred mouse strains, thus limiting analyses of the natural genetic variation of *Oas1b* found within populations. Genetic variation of immune genes plays an important role in infection outcome and contributes to host susceptibility and resistance (Ferris *et al.* 2013). As noted above, most wild derived inbred mouse strains produce a full-length *Oas1b* protein, whereas most if not all inbred mouse strains, including the strain of the reference genome, C57BL/6J, have a premature stop codon in *Oas1b* that links with susceptibility to WNV infection (Elbahesh *et al.* 2011; Mashimo *et al.* 2002, see Figure 6, Supplemental Material, Figure S1, and Table 1 and Table 2). To capture the genetic diversity of *Oas1b* for functional studies of WNV susceptibility, we evaluated its expression and linkage with WNV infection outcome and innate immune transcriptional signatures in the CC mouse population (Iraqi *et al.* 2012).

**Table 1:** QTL Loci

Innate Immune Activation QTL		
Chromosome and Region	Phenotype	Candidate Genes
Chr 5: 120–123 Mb	Weight loss (D12)	Oas1b* (see Table S1 for a complete list)

The genomic region identified in chromosome 5 where *Oas1b* was found. The QTL ranged between 2 and 3 Mb and includes *Oas1b*. QTL, quantitative trait locus; Chr, chromosome; D12, day 12 postinfection.

**Table 2:** Table of F1s screened for transcriptomics analysis

Cross	OASlb Status	Phenotype	Oaslb Origin	Dam	Sire
CC017×CC004	F/F	Asymptomatic	CAST/WSB	Functional	Functional
CC019×CC004	F/F	Asymptomatic	CAST/WSB	Functional	Functional
CC009×CC040	N/N	Symptomatic	N/A	Null	Null
CC006×CC007	N/N	Symptomatic	N/A	Null	Null
CC055×CC028	N/F	Symptomatic	PWK	Null	Functional
CC003×CC062	F/N	Asymptomatic	CAST/WSB	Functional	Null
CC030×CC061	F/N	Asymptomatic	CAST/WSB	Functional	Null

The F1s used in analysis along with their *Oas1b* status, and *Oas1b* founder origin. Gene expression changes were assessed against mock-infected congenic control mice for each cross. F/F, functional *Oas1b* alleles from Dam and Sire; N/N, homozygous null *Oas1b*; N/A, not applicable; N/F, heterozygous RIX lines where only the Sire has a functional *Oas1b* allele; F/N, heterozygous RIX lines where only the Dam has a functional *Oas1b* allele.

The CC is a MPP, which was generated to improve systems genetic research using the mouse as a model organism. The CC resource is composed of ∼70 independently bred, octo-parental recombinant inbred mouse strains (Srivastava *et al.* 2017), out of several hundred lines started (Shorter *et al.* 2017). Each CC line inherits haplotypes from three wild-derived (CAST/EiJ, PWK/PhJ, and WSB/EiJ) and five classical (A/J, C57BL/6J, 129S1/SvImJ, NOD/ShILtJ, and NZO/HiLtJ) inbred strains contributing both functional and nonfunctional OAS1b proteins into this MPP. Here, we use F1 crosses between CC strains, generated to understand complex and diverse phenotypic responses to virus infection (Rasmussen *et al.* 2014; Graham *et al.* 2016). The CC mice are an excellent model of WNV infection because their intron and exon structures in the *Oas* gene family are conserved (Choi *et al.* 2015). However, utilization of F1s allows sequences to differ between alleles at a locus, causing allelic heterozygosity between the wild-derived and classical inbred *Oas1b* sequences in this panel. Within this panel of F1s, we can generate classes of F1s based on their *Oas1b* alleles: functional *Oas1b* alleles from Dam and Sire (F/F), heterozygous RIX lines where only the Dam has a functional *Oas1b* allele (F/N), only the Sire (N/F), or neither parent has functioning *Oas1b* alleles (N/N). In previous work, we used the CC model to evaluate host responses to WNV and observed phenotypic variation that recapitulates the diversity in WNV susceptibility and outcomes observed in human populations (Graham *et al.* 2015, Green *et al.* 2016a, Green *et al.* 2016b).

In this study, we performed a coupled analysis of *in vivo* WNV infection, viral replication, host transcriptomics, and bioinformatics analyses to expand this genetic screen to identify regulatory loci (Table 1) and determine underlying transcriptional responses contributing to infection outcome. We screened 90 different F1 hybrids between CC strains infected with WNV to identify quantitative trait loci (QTL) contributing to disease phenotypes (Figure 1). To investigate the host response across F1s of different *Oas1b* haplotypes, transcriptomics was performed on the spleens of WNV-infected mice at 2, 4, 7, and 12 d postinfection. This transcriptional profiling revealed innate immune regulatory networks associated with the *Oas1b* haplotype. Our observations show that the CC is an important biomedical model for studies of human disease, and leveraging newly developed genomic resources in this model can improve our understanding of host responses controlling WNV infection and immunity.

**Figure 1:**
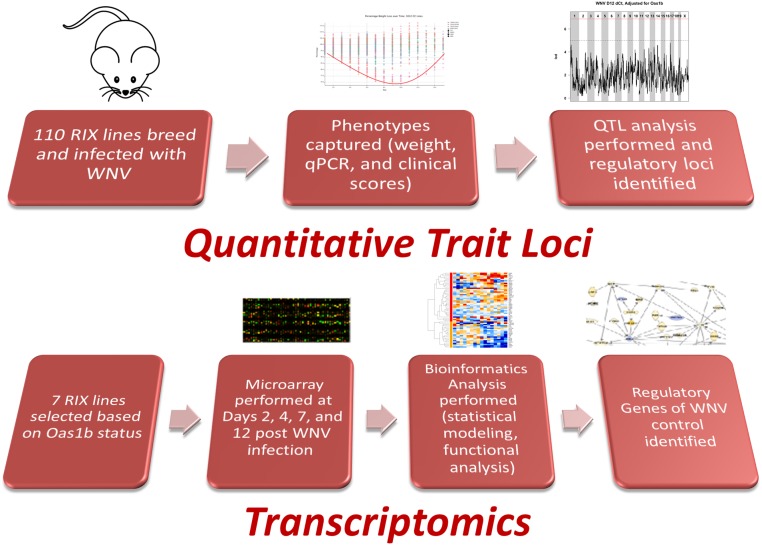
Analysis workflow. The analysis steps for generating innate immune regulatory networks and pathways influenced by *Oas1b*. qPCR, quantitative polymerase chain reaction; QTL, quantitative trait loci; RIX, recombinant inbred intercross; WNV, West Nile virus.

## Materials and Methods

### Mice and infection

F1s were bred at the University of North Carolina at Chapel Hill under specific-pathogen-free (SPF) conditions. The 6- to 8-wk-old male mice were transferred to the University of Washington and housed directly in a biosafety level 2 (BSL-2) laboratory within an SPF barrier facility. After a resting period, age- and sex-matched 8- to 10-wk-old mice were subcutaneously inoculated in the rear footpad with 100 PFU WNV TX-2002-HC (WN-TX). Mice were monitored daily for morbidity (percentage of initial weight loss) and clinical disease scores. Mice were then housed under BSL-3 conditions throughout the experiments, and tissues were processed under BSL-3 conditions. All animal experiments were approved by the University of Washington Institutional Animal Care and Use Committee. The Office of Laboratory Animal Welfare of the National Institutes of Health (NIH) has approved the University of Washington (A3464-01), and this study was carried out in strict compliance with the Public Health Service (PHS) Policy on Humane Care and Use of Laboratory Animals.

### Virus

WN-TX was propagated using previously described methods (Graham *et al.* 2016; Green *et al.* 2016). Viral stocks were generated using supernatants collected from infected Vero cell lines and stored at 80°.

### QTL analysis

To identify polymorphic host loci impacting phenotypic differences, we used a modified approach of the DOQTL bioconductor package (version 1.6) in R. The additive haplotype model was used in our analyses and in previous studies (Gatti *et al.* 2014). Under this model the regression equation is defined as:

$$\gamma_{i}=\sum_{k}x_{ik}\alpha_{k}\sum_{h=1}^{8}d_{ij}\left(h\right)\beta_{h}+\gamma_{i}+\varepsilon_{i}$$

where *Y*_i_ indicates the phenotype for animal *i*, *x* is the indicator of covariate (*k*) for animal (*i*), and α is the effect of covariate (*k*). *d_ij_*(*h*) is assigned to allelic dosage of the founder (*h*) at locus *j*. β*_h_* is a coefficient of the genetic effects in each founder. The second *Y_i_* adjusts for kinship and ε*_i_* for residual error in the animal (*i*). In order to generate additive allelic dosage probabilities for each F1, we generated eight-allele probabilities for each F1 with half of the probability for each allele coming from the inbred dam RI, and half coming from the sire RI, based on the MegaMUGA most recent common ancestor (MRCA) probabilities (http://csbio.unc.edu/CCstatus/index.py?run=FounderProbs) (Srivastava *et al.* 2017). The X-chromosome of these F1 males presents a special case, where the eight-allele probabilities are inherited fully from the dam RI. QTL genome scans were performed by regression on the day 12 weight change on genotype probabilities for each of the eight founder strains using R (see Supplemental Material for executable code). A random-effect term was included in the model to account for kinship among animals. A LOD score for each marker was calculated from the likelihood ratio comparing the regression model described above to a regression model without the founder genotype probabilities. The statistical significance of LOD scores was determined via a permutation test. A threshold of *p* < 0.05 was used to select significant associations.

DOQTL genotype probability files were generated with each animal having a unique genome probability entry. In this way, even animals from the same RIX would have individual (albeit identical) genome probability entries. This approach allowed us to assess both within- as well as between-strain phenotypic differences.

An adjusted *p*-value for each marker was not included because a permutation test was used to determine statistical significance of the LOD scores. The supplemental code provided contains hard coded significance thresholds to match the original analysis from the figures. Since there is some randomness to the permutation test, the threshold may display slightly different values if repeated.

### Identification of Oas1b allele status

We utilized haplotype probability reconstructions [based on a hidden Markov model, described in Iraqi *et al.* (2012) and Srivastava *et al.* (2017)] for each CC strain to identify which founder strain haplotype was present at the *Oas1b* locus. Previous work (Graham *et al.* 2015) had confirmed that *Oas1b* alleles from the CAST/Eij, PWK/PhJ, and WSB/EiJ strains had read-through codons relative to the reference genome (Keane *et al.* 2011), and this read-through variant is associated with protection from WNV infection. F1s were identified as *Oas1b* N/N if each parent had an *Oas1b* haplotype without the read-through variant (A/J, C57BL/6J, 129s1/SvImJ, NOD/ShILtJ, or NZO/HILtJ); *Oas1b* F/F if each parent had an *Oas1b* haplotype with the read-through variant (CAST/EiJ, PWK/PhJ, or WSB/EiJ); and *Oas1b* heterozygous if only one parent had the read-through variant.

### Oas1b sequence reconstruction

We integrated high confidence nonsynonymous SNPs from the Sanger mouse genomes project (www.sanger.ac.uk) (Iraqi *et al.* 2012; Oreper *et al.* 2017; Srivastava *et al.* 2017) of the eight CC founder strains with the annotated *Oas1b* exon sequence (Figure S1).

### Clinical scoring: CC F1s and their disease definitions

The clinical scoring system used to evaluate WNV-infected mice was as follows: 0, healthy mouse (baseline); 1, ruffled fur, lethargy, hunched posture, no paresis, and normal gait; 2, altered gait and limited movement in one hind limb; 3, lack of movement and paresis in one or both hind limbs; and 4, moribund.

### RNA extraction and quantitative PCR (qPCR) of WNV

Spleen and brain tissue were removed from mock- or WNV-infected mice. Half of the brain was homogenized immediately after harvest in 1 × PBS at 5500 RPM for 20 sec using a Precellys 24 machine and then centrifuged. Brain supernatant was added to TRI reagent (Ambion). Spleen tissues were stored in RNAlater (Ambion) and later homogenized in TRI reagent (Ambion). Total RNA was extracted using the Ribopure RNA Purification Kit (Ambion), with the addition of bromochloropropane (Acros Organics). RNA was converted to cDNA using the iScript Select cDNA Synthesis Kit (Bio-Rad). Using SYBR Green (Applied Biosystems) RT-PCR, WNV was quantified relative to GAPDH by probing cDNA with WNV-specific probes. qPCR results were recorded as fold change over mock-infected mice using the R statistical programming language (version 3.1). Results were loaded into Spotfire (http://spotfire.tibco.com, version 7.5.0.86) to produce box plots. Relative quantification was performed for WNV detection through qPCR. The forward and reverse primer used were: Primer WNV 1160F, 5′-TCA GCG ATC TCT CCA CCA AAG-3′ and Primer WNV 1229R, 5′-GGG TCA GCA CGT TTG TCA TTG-3′.

### Correlation matrix

Phenotypic results (clinical score, weight loss, and qPCR in spleen and brain) were loaded into R. Phenotypes were averaged by their genetic background (F1), tissue type, and time point. The Pearson correlation function (cor) was performed using R’s stats package and scores were displayed using the ggplot2 bioconductor package. For a complete listing of package information, data, and commands, please refer to the data reproducibility document in Github under correlation analysis.

### Determining outcome: symptomatic and asymptomatic

To quantify disease outcome, we used weight loss and clinical scoring to segregate the F1s into two broad pathogenic phenotype categories: asymptomatic or symptomatic. Three animals were assessed for each F1 and outcome was based on at least one mouse out of three meeting weight loss or clinical score criteria at any time point. Symptomatic were defined as having weight loss > 10% of original preinfection weight, clinical score > 1, and/or death, whereas asymptomatic was defined as having weight loss < 10% of original preinfection weight, clinical score of 0 or 1, and no death.

### Affymetrix target preparation and microarray hybridization

RNA spleen samples were prepared for whole-transcriptome expression analysis using the WT PLUS Reagent Kit following the manufacturer’s recommended protocol (Affymetrix, Inc.). Next, 100 ng total RNA was used to prepare the hybridization-ready targets. Individual sense-strand DNA targets were randomized and hybridized to Mouse Gene 2.1 ST 96-Array Plates (Affymetrix, Inc.) using the GeneTitan Multi-Channel Instrument for hybridization, staining, and washing of arrays, as well as for scanning. Quality control (QC) metrics for hybridization, labeling and sample quality were generated using the Affymetrix Expression Console (version 1.3.187) software. All samples passed QC criteria.

### CC-probe masking

To ensure that all Affymetrix probes were identified across all CC lines, previously described masking techniques were applied based on the CC founder data (Graham *et al.* 2016; Rasmussen *et al.* 2014). To mask out inconsistent Affymetrix probes, we used the oligomask R package designed for CC mouse data (Bottomly *et al.* 2014). Oligomask uses VCF files and the oligo package to filter probes prior to normalization and statistical analysis.

### Transcriptomic analysis

Samples were screened for QC and outlier detection using the Affymetrix expression console using boxplots, as well as multi-dimensional scaling analysis and interarray correlation plots using the R statistical programming language (https://www.r-project.org, version 3.1), Bioconductor (version 2.13), and packages oligo (1.32.0), oligomask (https://github.com/dbottomly/oligoMask), limma (3.28.4), and corresponding dependencies. Background correction, normalization, probe masking, and probe summarization were performed with RMA (Robust Multichip Average) within the oligomask package. The sva package (3.20) in R was used to correct for plate batch effects. Differential expression (DE) analysis was performed using the limma bioconductor package (see statistical analysis under supplemental wiki in Github). A statistical cutoff of > 1.5-fold change over mock with a Benjamini–Hochberg-adjusted *p*-value < 0.05 was applied. The union of all DE genes for at least one time point in at least one F1 was first obtained and then filtered for immune genes. Immune genes were selected as genes identified in immune-related canonical pathways from Ingenuity pathway analysis (IPA). This list of immune-related genes was used for the global heatmap (Figure 4). The ABC (ATP binding cassette) and oxysterol-binding proteins were added to the screen to observe their correlation with other known *Oas1b* binding partners (Courtney *et al.* 2012). The heatmap was created using the heatmap.2 function in gplots (3.0.1) in R and bioconductor. A data report containing executable code can be found in Github under transcriptional analysis. Data were deposited in the GEO repository (access number GSE91003).

### Mock correlation

Due to infection and sample collection scheduling, most mock samples were collected at either 2 or 12 d postinfection per F1. To ensure that this had no impact on normalized transcriptional activity, probe-masked expression data were compared in mocks for two separate F1s, CC(041x012)F1 and CC(004x011)F1, at days 12 and 28 postmock-infection (data not shown). Pearson correlation in R gave scores (0.9688 and 0.9687) that confirmed high correlation in the expression between mocks of the same F1s at different time points.

### Coexpression heatmap

Coexpression was performed only on genes that were determined to be statistically significant from the differential expression analysis (threshold: log2 fold change ≥ 0.58 and FDR ≤ 0.05) in at least one comparison and considered immune-related (see transcriptional analysis). Pearson correlations were run on the union of log_2_FC using the WGCNA and heatmap.2 bioconductor packages in R (Loraine *et al.* 2015; Gentleman *et al.* 2004; Smyth 2004)

### Pathway and regulator analysis

A list of statistically significant, differentially expressed genes (threshold of significance of a > 1.5-fold change over mock with a Benjamini–Hochberg-adjusted *p*-value ≤ 0.05) were uploaded into IPA for core analysis to identify enrichment of biological pathways. The tools produce a list of known biological functions with an enrichment score (−log *p*-value) determining how significant those genes are to each function, and an activation *z*-score that tells the proposed activation of that pathway (activated or inhibited).

The *z*-score is based on knowledge of expression changes (and functions) in the Ingenuity knowledge base (Krämer *et al.* 2014). After the core analysis was performed in IPA, it was exported into Spotfire (version 7.5.0.86). Plots were created conservatively based on the following criteria: each biological pathway needed a −log *p*-value ≥ 1.30 (corresponds to *p*-value ≤ 0.05), two or more genes in each pathway, and had to produce an activation *z*-score. For the regulator effects network, the F/F, CC(019x002)F1 was analyzed for expression changes at 2 d postinfection and the top scoring network (antiviral response, 63% known regulators) was observed. Network generation was performed within IPA.

### Innate immune networks

Genes detected in linear modeling and correlation analyses were loaded into IPA and networks were selected based on enrichment for immune-related pathways. The F/F network was based on the top two highest scoring networks (immunological disease and inflammatory response with network scores 36 and 33). The F/N network was based on immune and inflammatory functions (network score 35). The N/N network was built on functions in cell-to-cell signaling (network score 27). Protein–protein interactions from the Ingenuity knowledge base were used to connect ABCF3 and ORPL1 with the transcriptional data. This was done using the network build and connect tools in IPA. Network data were then exported into cytoscape (version 3.1.1) to make custom network figures.

### Data availability

A complete description of the data and methods can be found on the manuscript’s Github page (https://github.com/greener98103/oas1b/wiki) and in the supplemental file titled “Green_Oas1b_data_policy_documentation.” This file includes information on strains/lines, variant files from Sanger, and marker files [MUGA platform (Morgan *et al.* 2015)]. The data document also contains detailed descriptions, file locations, and links to executable code for QTL, transcriptional, and correlation analyses. Gene expression data are in GEO with the accession number GSE91003. The authors state that all data necessary for confirming the conclusions presented in the article are represented fully within the article.

## Results

### A QTL identified in chromosome 5 found in day 12 weight loss

**Figure 2:**
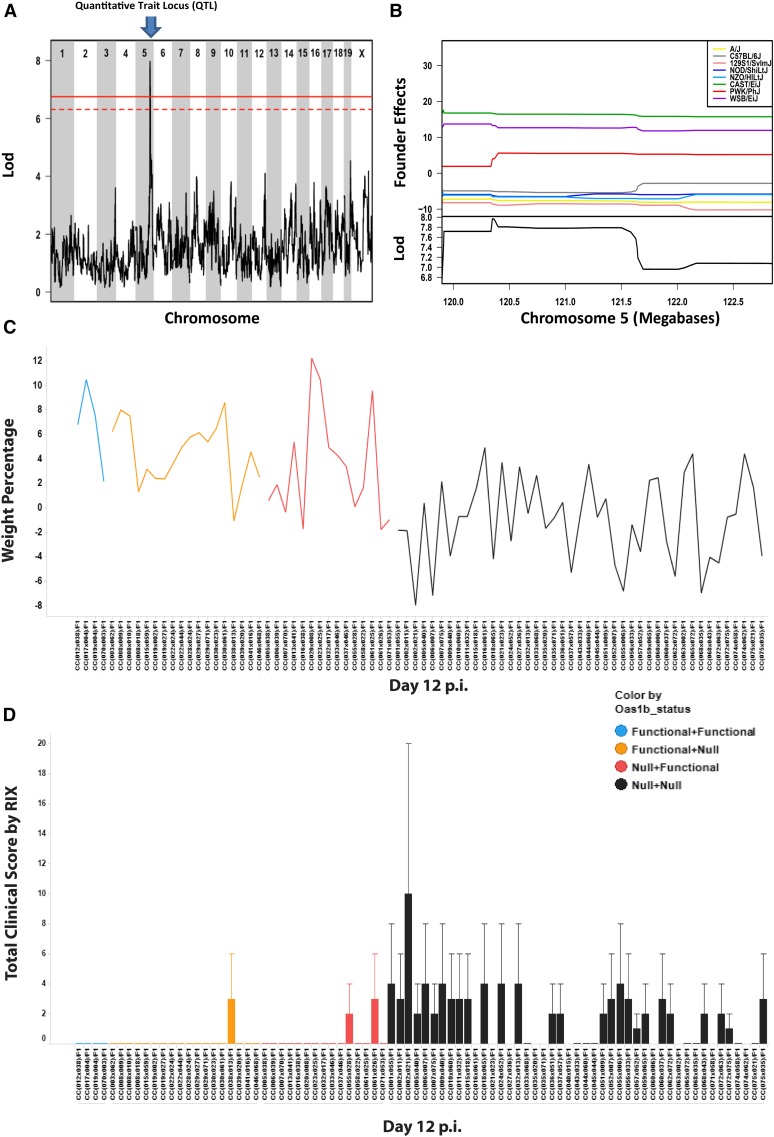

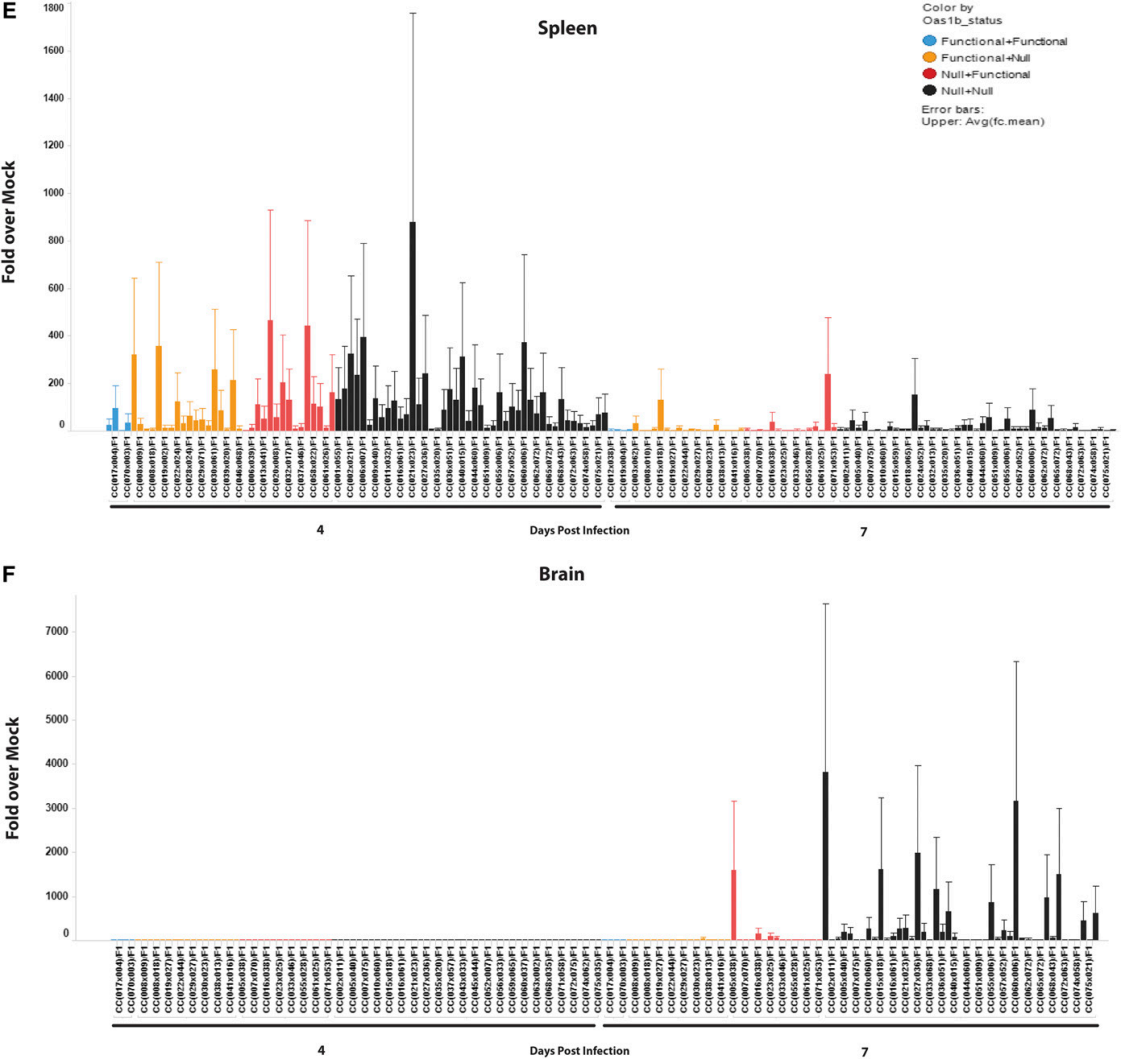
WNV QTL. (A) Genome scan of 90 F1s from day 12 weight loss with a significant QTL peak on chromosome 5. The top row indicates chromosome number and the *y*-axis is the LOD score. The LOD is the degree of concordance for a genetic marker for a phenotype (weight loss at day 12). The solid red line is *p*-value = 0.05 and the dotted line is *p* = 0.1. (B) The upper image is the contribution of each founder in the QTL (founder effects), where the population average is zero and each colored line represents a different founder’s predicted effect of one allelic copy on an individual’s phenotype (weight percentage at day 12). The lower image shows the LOD score in the lower *y*-axis. The *x*-axis shows the locus in megabases at chromosome 5. Each colored line represents a different founder’s predicted effect on phenotype. The positive alleles on the top show founder lines contributing to greater than the population mean, and the negative values contributing to less than the population mean. (C) Weight change at day 12 p.i. The *y*-axis represents weight percentage (gain or loss) across all RIXs. The *x*-axis shows the F1 identity and the color indicates *Oas1b* haplotype. Blue = F/F, orange = F/N, red = N/F, and black =N/N. (D) Total clinical score per F1 at day 12 p.i. Clinical score was determined as follows: 0, healthy mouse (baseline); 1, ruffled fur, lethargy, hunched posture, no paresis, and normal gait; 2, altered gait and limited movement in one hind limb; 3, lack of movement and paresis in one or both hind limbs. qPCR of WNV virus in the spleen (E) and brain (F) at days 4 and 7 p.i. The *y*-axis shows fold change over mock and the *x*-axis shows each F1 background and their days p.i. Each color represents *Oas1b* haplotype. Blue = F/F, Orange = F/N, Red = N/F, Black = N/N. LOD, log odds ratio; p.i., postinfection; qPCR, quantitative polymerase chain reaction; QTL, quantitative trait loci; RIX, recombinant inbred intercross; WNV, West Nile virus.

We conducted QTL mapping across our panel of 90 F1s (Table 1), and identified an approximate 2.5 Mb region spanning from 120–122.5 Mb on chromosome 5 (here termed the host response to WNV, region 1, or *Hrw1*) (Figure 1A). HrW1 impacted day 12 weight loss (Figure 2, A and C). The *Hrw1* aggregate peak contained 70 genes and microRNAs (excluding predicted genes). The genes under this locus include all the murine *Oas* genes as well as genes associated with biological pathways including actin cytoskeleton signaling, CD28 signaling in T helper cells, and integrin signaling (Table S1 and Table S2). The QTL peak sits over *Oas1b*. Based on previous mouse studies (Suthar *et al.* 2013; Mashimo *et al.* 2002; Ferguson *et al.* 2008; Kajaste-Rudnitski *et al.* 2006), *Oas1b* is a known driver of disease phenotypes. To determine which of the eight strains in the CC were influencing the loci, we next looked at the founder effects driving this QTL.

### Loci founder effects

As the CC is derived from eight founder strains, QTL can contrast any combinations of these founder haplotypes. Figure 2B shows the estimated phenotypic effect of each founder strain haplotype at the locus on day 12 weight. Haplotype effects are determined as the deviation from the population mean due to one copy of that founder allele at the locus. In this way, we can see that the wild-derived inbred strains CAST/EiJ, PWK/PhJ, and WBS/EiJ ameliorate weight loss (higher value = less weight lost), whereas the five classic inbred strain alleles show enhanced weight loss (lower effects). These results are consistent with a previously reported analysis of *Oas1b* (Courtney *et al.* 2012) and the Sanger mouse genomes project (Keane *et al.* 2011), which show that classical strains have an early stop codon, whereas wild-derived founders encode a fully functional protein. These different founder contributions yield a range of complex phenotypes seen in the F1s. Previous CC studies showed that F1s heterozygous for *Oas1b* did not guarantee protection (Graham *et al.* 2016) and there were likely other immune factors beyond *Oas1b* driving WNV infection outcome. To get a global view of WNV pathogenesis in the CC, we studied disease responses by haplotype.

### Contribution of Oas1b in preventing viral growth and neuroinvasion

Guided by the observed allele effects, and the presence of the truncated stop codon in *Oas1b*, we binned our F1s into four classes: F/F (any F1 containing CAST/EiJ, PWK/PhJ, and/or WSB/EiJ alleles), N/N (any F1 containing A/J, C57BL/6J, 129S1/SvImJ, NOD/ShiLtJ, and/or NZO/HILtJ), and the two reciprocal heterozygous classes F/N (a maternally inherited functional allele and a paternally inherited defective allele) and N/F (a maternally inherited defective allele and a paternally inherited functional allele). We measured a variety of WNV disease response phenotypes and assessed their differences between these classes. F/F and heterozygous F1s displayed similar fluctuations in weight at day 12 postinfection (Figure 2C) compared to N/N F1s. Of the heterozygous F1s, N/F showed a slightly higher distribution in weight at day 12 compared to F/N haplotype mice. Clinical scores were also evaluated at day 12 (Figure 2D). Clinical score was calculated by animal’s appearance and physical function characteristics (ruffled fur, lethargy, hunched posture, and no paresis) and movement (see *Materials and Methods*). The only mice that developed overt clinical scores associated with WNV disease were from the N/N *Oas1b* F1s.

WNV is a neuroinvasive virus that first replicates in the spleen and then progresses to the central nervous system in a manner controlled by the innate immune response (Suthar *et al.* 2013; Elbahesh *et al.* 2011). To assess viral growth and neuroinvasion, we measured the levels of WNV virus in the spleen and brain using qPCR. An inability to effectively control WNV growth in the periphery (such as the spleen) facilitates viral production and neuroinvasion (Suthar *et al.* 2013; Green *et al.* 2016a; Fredericksen 2014). qPCR results showed that F1s without functioning *Oas1b* alleles displayed an increased viral load in the spleen at day 4 (Figure 2E). By day 7, this lack of viral control resulted in WNV neuroinvasion characterized by virus in the brain (Figure 2F). There were lower levels of virus in heterozygous F1s in both the spleen at day 4 and in the brain by day 7. The accumulation of virus in N/N was statistically significant in the brain compared to heterozygous F1s (*p*-value = 0.0005) across all time points (2, 4, 7, and 12 d postinfection). Viral accumulation was not statistically significant in the spleen, but we observed a correlation between viral qPCR and clinical score in the N/N and heterozygous F1s (see Figure S2 and Github).

### Distribution of infection response

**Figure 3:**
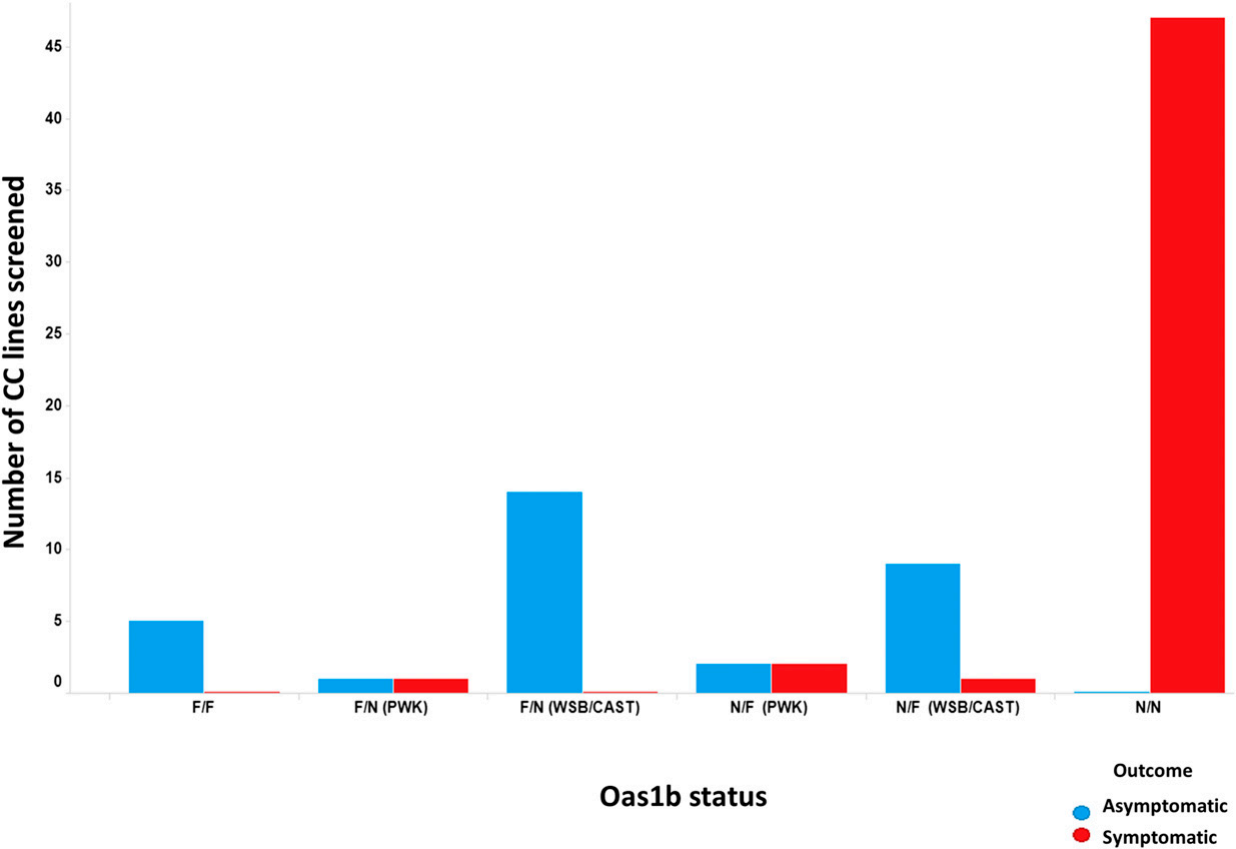
Disease outcome under *Oas1b* functionality. The number of F1s summarized by disease outcome. Outcome was determined as symptomatic (loss of 10% or greater body weight or succumbed to WNV infection) or asymptomatic (fought off infection and never lost 10% weight). Each group of mice is binned according to their *Oas1b* status. The heterozygous F1s are shown by their disease outcome and their allelic founder contribution. F/N(PWK) means the F1 contains at least one PWK allele (Null/PWK or PWK/Null). F/N(WSB/CAST) means the F1 contains at least one WSB/CAST allele. The colors indicate each’s F1’s outcome (blue = asymptomatic and red = symptomatic). CC, Collaborative Cross; WNV, West Nile virus.

From the 90 F1s used for QTL mapping based on weight loss, we evaluated 83 F1s because they had clinical metrics of susceptibility to WNV infection. Responses of each line were captured and an outcome was determined as asymptomatic or symptomatic. For a line to be considered symptomatic, infected animals had to exhibit a clinical score > 1 or a > 10% loss in weight since the time of infection (see *Materials and Methods*). We evaluated infection outcome based on haplotype (Figure 3). Out of the 83 F1s screened, 47 of them (56%) were symptomatic and did not contain full-length *Oas1b* alleles. Only five F1s lines were asymptomatic with F/F alleles (6%), and zero lines with F/F alleles were symptomatic. Likewise, no F1s with N/N alleles were determined to be asymptomatic. F/F *Oas1b* alleles provided complete protection against WNV clinical disease. Of the asymptomatic heterozygous F1s, (F/N and N/F), 16 were F/N (19%) compared to 14 N/F (17%). We only observed four symptomatic N/F F1s (5%) compared to one asymptomatic F/N F1 (1%). We also observed a relationship between viral load and disease phenotypes (see Figure S2).

To identify differences between the heterozygous lines, we looked at allelic contributions among the eight founder strains. There was a subtle difference in disease outcome among the heterozygous lines but this was likely driven by lines containing an *Oas1b* allele from the WSB/EiJ or CAST/EiJ founder strains. F1s that contained the *Oas1b* allele from the PWK/PhJ founder strain appeared to only provide moderate protection compared to either the WSB/EiJ or CAST/EiJ alleles (see *Materials and Methods*). These observations support previous studies implying a major role for *Oas1b* in protection against WNV disease in mice, although this is the first time a comparison has been made between heterozygous F1s and their *Oas1b*’s founder effects.

To reveal innate immune signatures driving WNV susceptibility, we performed a multi-tier bioinformatics analysis (see Figure 1) involving genome-wide microarray analysis on a set of F1s with different *Oas1b* haplotypes (F/F, F/N, N/F, and N/N), focusing on revealing the profile of immune-related genes in the spleen at days 2, 4, 7, and 12 post-WNV infection as compared to mock-infected congenic control mice (Table 2). We centered our transcriptional profiling assessment on the spleen to better understand peripheral immune programming and possible linkage with the control of viral neuroinvasion.

### Coregulatory patterns between Oas1b haplotypes

**Figure 4:**
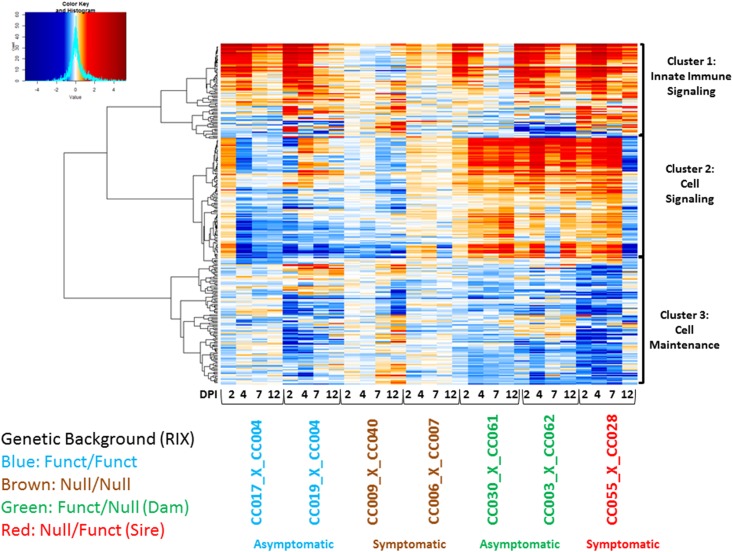
Heatmap of immune disease modules. Coexpression in spleen at days 2, 4, 7, and 12 postinfection. The heatmap represents differential expression of 450 genes across seven F1s with different *Oas1b* functionality (F/F, N/N, F/N, and N/N). Expression is shown as log2(FC) WNV infected relative to mock. Red marks genes that are upregulated, blue for downregulated, and white represents no log2(FC). The *y*-axis shows genes that coregulate based on their directionality and magnitude. The *x*-axis shows the days postinfection, haplotype, and disease outcome according to the legend. Listed on the right as cluster 1: innate immunity, cluster 2: Cell maintenance, and cluster 3: cell signaling. log2(FC), log2 fold change; RIX, recombinant inbred intercross; WNV, West Nile virus.

We performed a transcriptional correlation analysis using the union of statistically significant genes across F1s with different haplotypes (Figure 4). Since the genetic backgrounds among F1s vary and those without *Oas1b* produced a large amount of differentially expressed genes (especially in later time points due to increased illness), we focused on statistically significant immune-related genes. Correlation analysis identified three distinct gene clusters. Functional analysis was performed to summarize the clusters into known biological functions. Genes within cluster 1 are largely involved in innate immunity, genes within cluster 2 include those involved in cellular signaling, and cluster 3 genes include a role in cell maintenance (Figure 4).

Genes in cluster 1 (innate immune signaling) include several molecules involved in RLR signaling, IFN responses, and death receptor signaling. The coregulated gene module contained ISGs, *Myd88*, *Oas2*, *Oas3*, *Stat2*, and *Tlr7*. Cluster 1 was also enriched for IFN regulatory factors (*Irf3*, *Irf7*, and *Irf9*). Expression of genes in this cluster appeared to be activated across all F1s, although not all met statistical significance. Within the F/F F1s, activation of this cluster was highly induced only until day 4. This profile is different from the heterozygous F1s, especially the symptomatic N/F F1 CC(055x028)F1, which remained induced through day 12. The *Oas* genes also appear in cluster 1. These heatmap and qPCR results display an early termination of innate immune signaling in the asymptomatic lines that could be influencing disease outcome. Expression changes in the second cluster (cell signaling) are primarily influenced by the three heterozygous F1s. The genes in this module are associated with the cell cycle, DNA replication, DNA repair, growth, and proliferation. This cluster/gene module showed little variation across heterozygous F1s and was sparsely suppressed in the F/F F1s. Expression was inverted at day 12 between the asymptomatic and symptomatic F1s (F/N and N/F, see Table 2), suggesting that this subcluster links with viral infection and differential WNV disease.

The expression changes found in CC(055x028)F1 between days 7 and 12 postinfection correspond with viral load. At day 12 postinfection, we found an increase in viral load in the brain as measured by qPCR (Figure S6). The gene expression changes between days 7 and 12 postinfection could mark viral spread and subsequent neuroinvasion.

The coexpressed genes found in the third cluster (cell maintenance) are involved in cell/tissue morphology and leukocyte extravasation (diapedesis). Genes in this biological pathway are suppressed in expression relative to mock-infected controls, implying that leukocyte migration is less active during infection in CC(009x040)F1 (N/N) and CC(055x028)F1 (F/N). Inhibition of leukocyte migration (and supporting chemokines) to infection sites could destabilize protection against WNV in these F1s.

To validate select innate immune gene expression, we performed qPCR on two virus-inducible genes: tetratricopeptide repeats 1 (*Ifit1*) and IFNβ (*Ifn*β*1*) using the F1s shown in our heatmap and in Table 2. *Ifit1* and *Ifn*β*1* are IRF3 target genes induced upon WNV infection; *Ifit1* is also an ISG and is highly expressed in response to type 1 or type 3 IFNs. Not surprisingly, *Ifit1* induction was highest at day 2 postinfection, indicative of IRF3 activation and the onset of IFN signaling (Figure S3 and Figure S4). All F1s appeared elevated for *Ifit1* but it was highest in F/F CC(019x004)F1. *Ifit1* decreased by day 4 post-WNV infection, indicating that viral replication was being controlled. The *Ifit1* elevation in N/N F1s at day 2 post-WNV infection indicated induction of IFN production. *Ifn*β*1* appeared low across most of the F1s in the spleen except the one N/N F1 at day 2 post-WNV infection. This IFN response was also seen in the brain in the N/N along with virus at day 7 and suggests that the F/F F1 was protective from the neuroinvasion (Figure S4). In summary, the gene expression profile of both F/F and N/N *Oas1b* haplotypes revealed expressed IFN signaling at day 2, but this alone did not link with protection in the N/N line.

### Transcriptional analysis of genes within the Hrw1 locus

**Figure 5:**
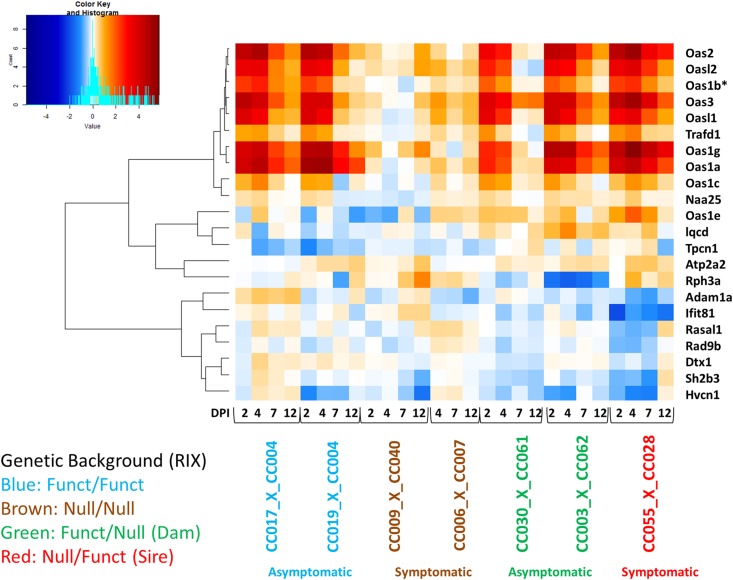
Heatmap of QTL genes. Coexpression in spleen at days 2, 4, 7, and 12 postinfection. The heatmap represents differential expression of 21 genes found under the QTL across seven F1s with different *Oas1b* haplotype (F/F, N/N, F/N, and N/N). Expression is shown as log2(FC) WNV infected relative to mock. Red marks genes that are upregulated, blue for downregulated, and white represents no log2(FC). The *y*-axis shows genes that coregulate based on their directionality and magnitude. The *x*-axis shows the days postinfection, haplotype, and disease outcome according to the legend. log2(FC), log2 fold change; QTL, quantitative trait loci; RIX, recombinant inbred intercross; WNV, West Nile virus.

To determine if expression differences in the *Oas* family members and other genes within the *Hrw1* locus on chromosome 5 (Figure 5) linked with WNV infection outcome, we applied a targeted transcriptional analysis within the locus. The genes that flank the *Oas* cluster (*Dtx1* and *Rph3a*) were identified as expressed compared to mock-infection in our heatmap. There are several genes whose expression was reduced (downregulated) independently of *Oas* signatures in the heterozygous F1s. Interestingly, *Rasal1* appeared downregulated in CC(055x028)F1, which was previously observed with elevated viral load in the spleen and brain (Scherbik *et al.* 2006), and this finding was confirmed with qPCR in the spleen at day 4 postinfection (see *Materials and Methods*). *Rasal1* is an inhibitory regulator of the Ras-cyclic AMP pathway and is involved in dendrite formation (observed in melanocytes), intercellular signal transduction, and GTPase activity, and could be implicated in WNV disease. We also evaluated the expression of other *Oas* gene family members and found that the F1s with the largest induction of *Oas* genes were CC(017x004)F1 (F/F), CC(019x004)F1 (F/F), and CC(055x028)F1 (N/N). *Oas1b* is included in Figure 5 and was induced by WNV infection to be differentially regulated compared to mock, but was not always statistically different across infection time points after multiple statistical comparisons. *Oasl1* (*Oas-like gene 1*), *Oas3*, and *Oas1a* were the most highly induced (upregulated) *Oas* gene family members across the panel. *Oas1a* and *Oas1g*, which are influential in viral detection and the innate immune response, were the next highest in expression. *Oasl1* has been shown to regulate IRF7 production (Lee *et al.* 2013). *Oas3* was found upregulated in each F1 we screened using qPCR and transcriptomics in at least one time point except for N/N CC(090x040)F1, which had low gene expression levels. The importance of *Oas3* in virus response is corroborated by a previous study that found that OAS3 displayed a higher affinity for dsRNA than OAS1 or OAS2, which indicated its contributive role in the antiviral response (Li *et al.* 2016). Importantly, none of the significant *Oas* genes were suppressed or “downregulated” in our analyses. We validated our findings with qPCR in *Oas* target genes *Oas1g*, *Oas2*, and *Oas3*. Thus, our analysis of *Oas* gene family members is supportive of *Oas* genes playing a significant role in antiviral immunity.

### Oas1b haplotype distinguishes gene expression modules

**Figure 6:**
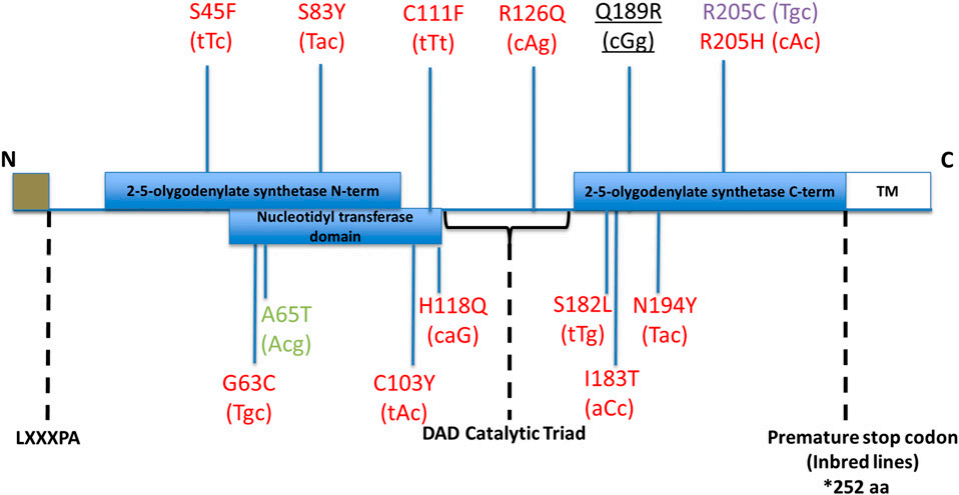
*Oas1b* protein. SNPs that impact aa sequence identified over annotated (Uniprot) functional domains. Changes in aa are coded relative to the reference sequence, which are identical for the five classical inbred founder strains, and changes are assigned to strains (PWK/PhJ = red; WSB/EiJ = purple; WSB/EiJ and Cast/EiJ = green; and PWK/PhJ, WSB/EiJ, and CAST/EiJ = underlined black). aa, amino acid; DAD (Motif); SNP, single nucleotide polymorphism; TM, transmembrane domain.

We conducted pathway analysis to assess the biological differences of significant genes in F1s of differential *Oas1b* haplotype. As shown in Figure 6, the Sanger sequencing revealed several polymorphisms within functional domains of the full-length *Oas1b* protein among alleles encoded in the PWK/PhJ, WSB/EiJ, and CAST/EiJ outbred parental CC strains compared to the C57BL6/J reference strain, while all of the classical inbred founder strains encoded a stop codon within the OAS1b C-terminal domain. We first focused on the presence and absence of full-length *Oas1b* to reveal differences in gene module expression between the two groups.

**Figure 7:**
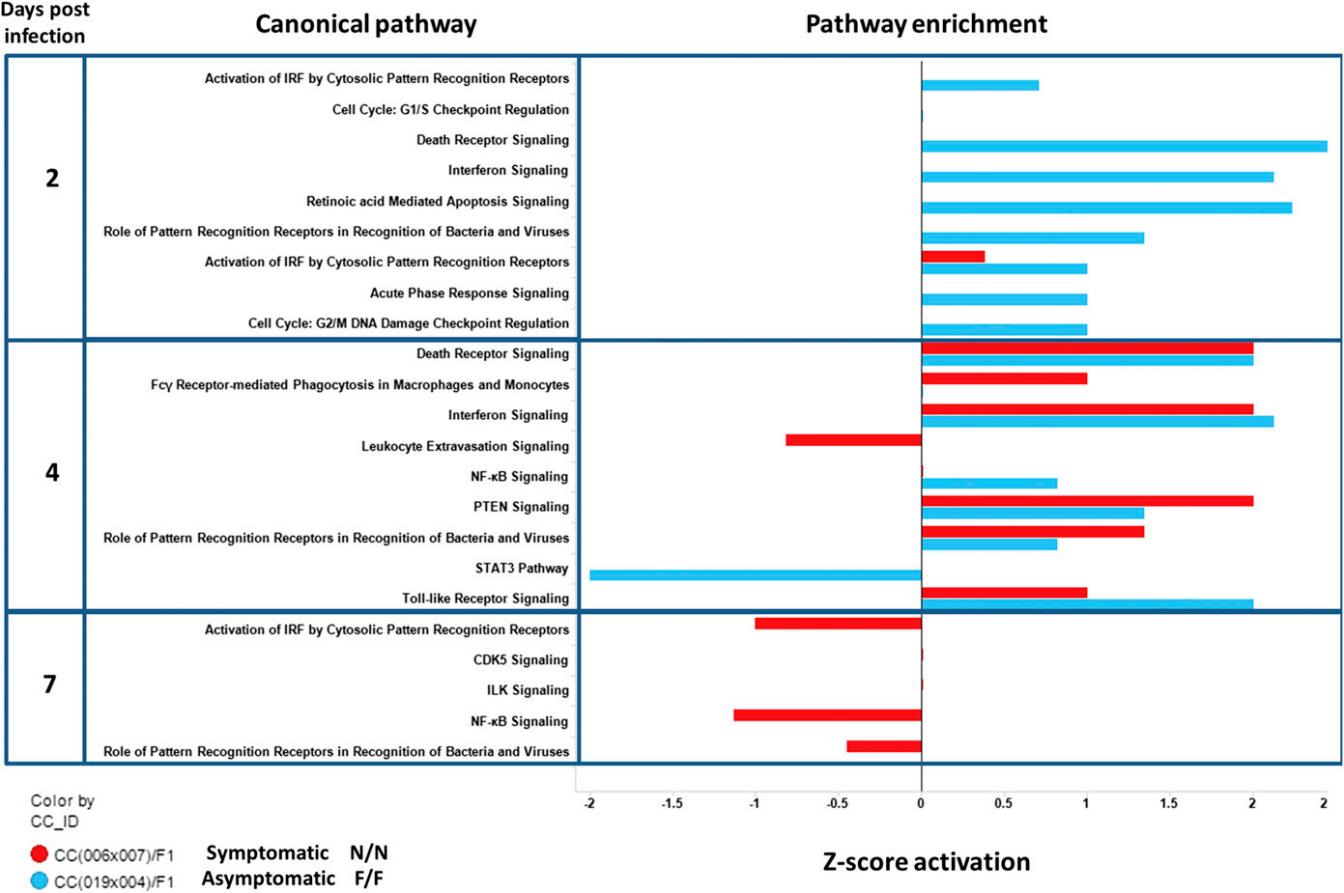
Pathway analysis in the presence (F/F, Functional + Functional Oas1b) and absence (N/N, Non-functional + Non-functional Oas1b) of functioning *Oas1b*. A bar plot quantifying enrichment of biological pathways across F1s and time points (2, 4, and 7 days) postinfection. The *y*-axis indicates time point and biological pathway. The *x*-axis shows the activation *z*-score for a pathway. The *z*-score is based on literature findings and determines the magnitude of gene regulation for a pathway. Values to the left indicate that pathway is inhibited and values to the right indicate that pathway is activated. The colors in the bar plot mark the different F1 backgrounds [blue = CC(009x040)F1 and red = CC(006x007)F1].

The immune response at day 2 postinfection (Figure 7) was impacted by *Oas1b* status. Immune pathways in F/F F1s were predominantly activated between days 2 and 4 postinfection. Aside from low level viral recognition, the N/N CC(006x007)F1 did not show significant activation in immune pathways until day 4 postinfection. By day 4 postinfection, several pathways were expressed in parallel, including pathways involving death receptor signaling, IFN response, toll like receptors, and viral recognition receptors, thus, marking the onset of the innate immune response. In contrast, STAT3 signaling was downregulated by day 4 postinfection in the F/F asymptomatic F1s, reflecting differential STAT3 signaling activity between the two groups. By day 7 postinfection, the N/N F1 profile marked a reduced transcriptional response within innate immune signaling pathways including NF-κb, and pattern recognition receptor signaling, possibly reflecting the progression of WNV infection out of the spleen onward to the CNS.

**Figure 8:**
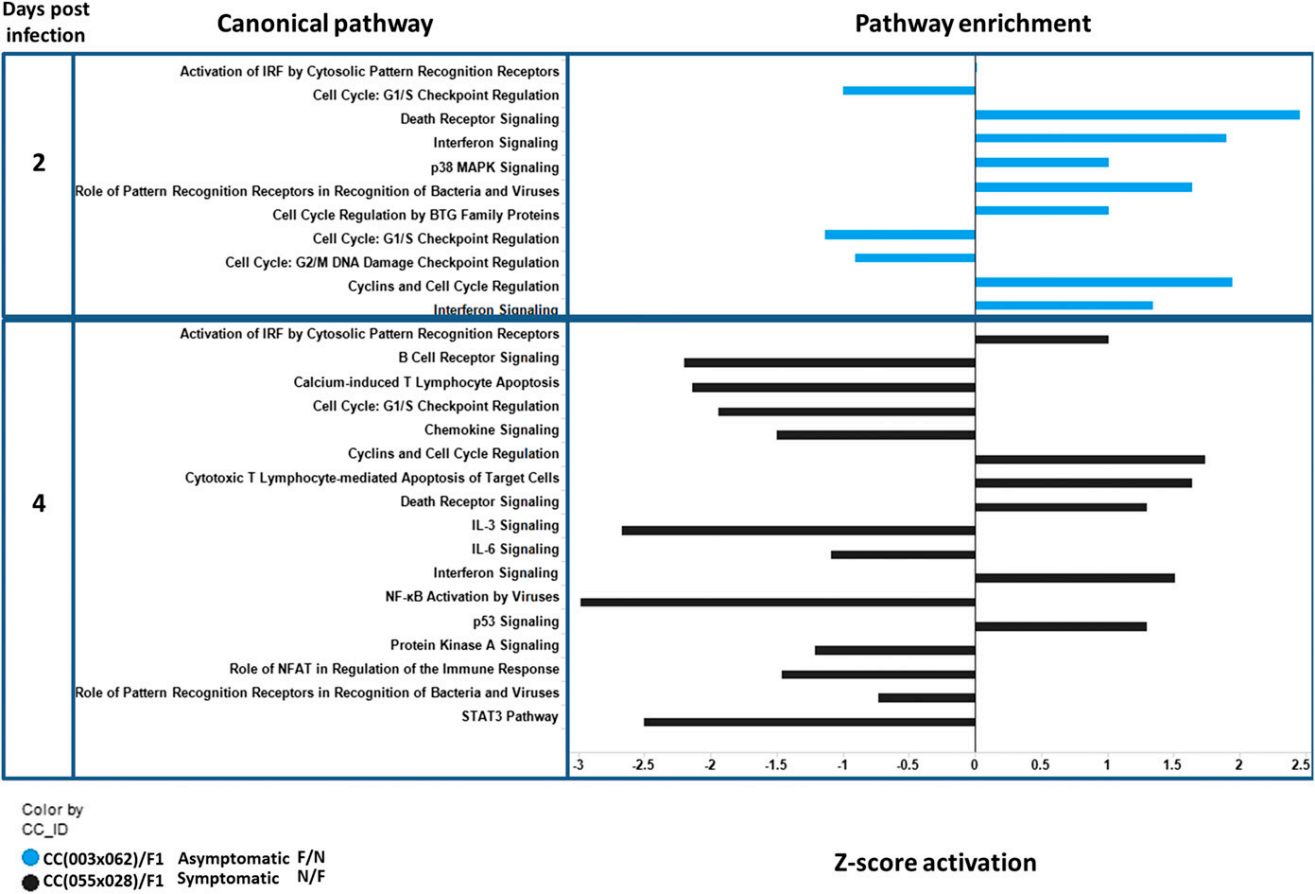
Pathway analysis in the heterozygous *Oas1b* F1s at day 2 and 4 post-WNV infection. A bar plot similar to day 4 showing enriched biological pathways. The *y*-axis indicates the haplotype, time point, and biological pathway. The *x*-axis shows the activation *z*-score for a pathway. Values to the left indicate that pathway is inhibited. Values to the right indicate that pathway is activated. The colors in the bar plot mark the different F1 backgrounds [blue = CC(003x062)F1 and black = CC(055x028)F1]. CC_ID, Collaborative Cross identifier.

To assess gene expression pathways among *Oas1b* heterozygous F1s, we concentrated on the day 2 and 4 postinfection time points (Figure 8). These pathway analyses compared the downstream effects of *Oas1b* between symptomatic CC(055x028)F1, whose *Oas1b* allele is derived from PWK/PhJ, *vs.* asymptomatic F/N CC(003x062)F1, whose *Oas1b* is from WSB/EiJ. We observed differences in G1/S checkpoint cell cycle pathways between CC(055x028)F1 and CC(003x062)F1. The pathway was downregulated in the asymptomatic line, but had very weak induction in the symptomatic line, possibly marking differential pathology among the lines. At day 2 postinfection, N/F CC(055x028)F1 had reduced expression of p53 signaling wherein the other heterozygous lines showed differential p53 module expression and cell signaling response modules at day 4 post-WNV infection. We observed more extensive gene module regulation in the *Oas1b* heterozygous mice overall.

### Innate immune regulatory networks

**Figure 9:**
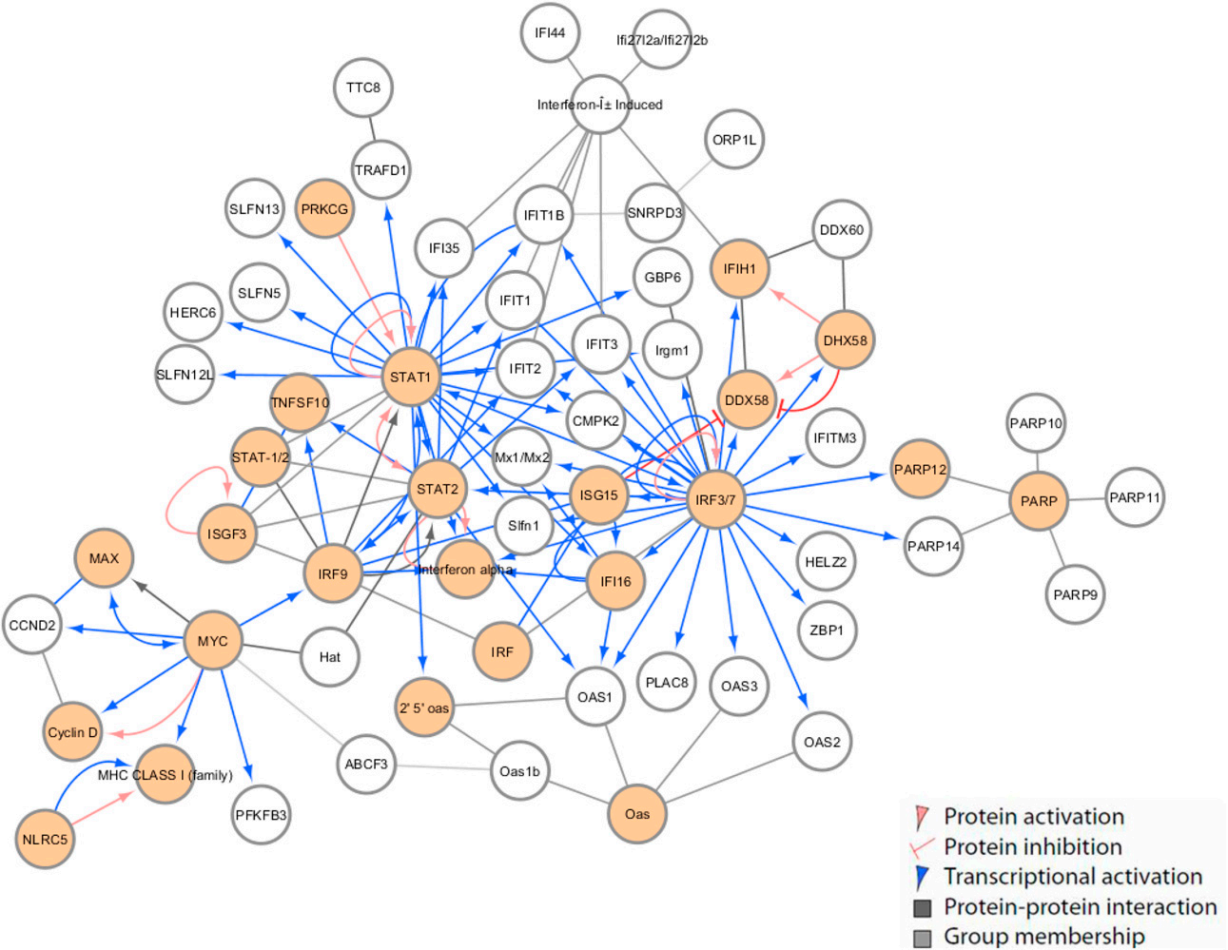
Innate immune regulation network [*Oas1b* allele (F/F)]. Innate immune regulatory network identified in F1s with F/F *Oas1b* alleles. Each node represents a gene with different colored lines showing types of connectivity. Filled-in nodes are target genes from the dataset.

**Figure 10:**
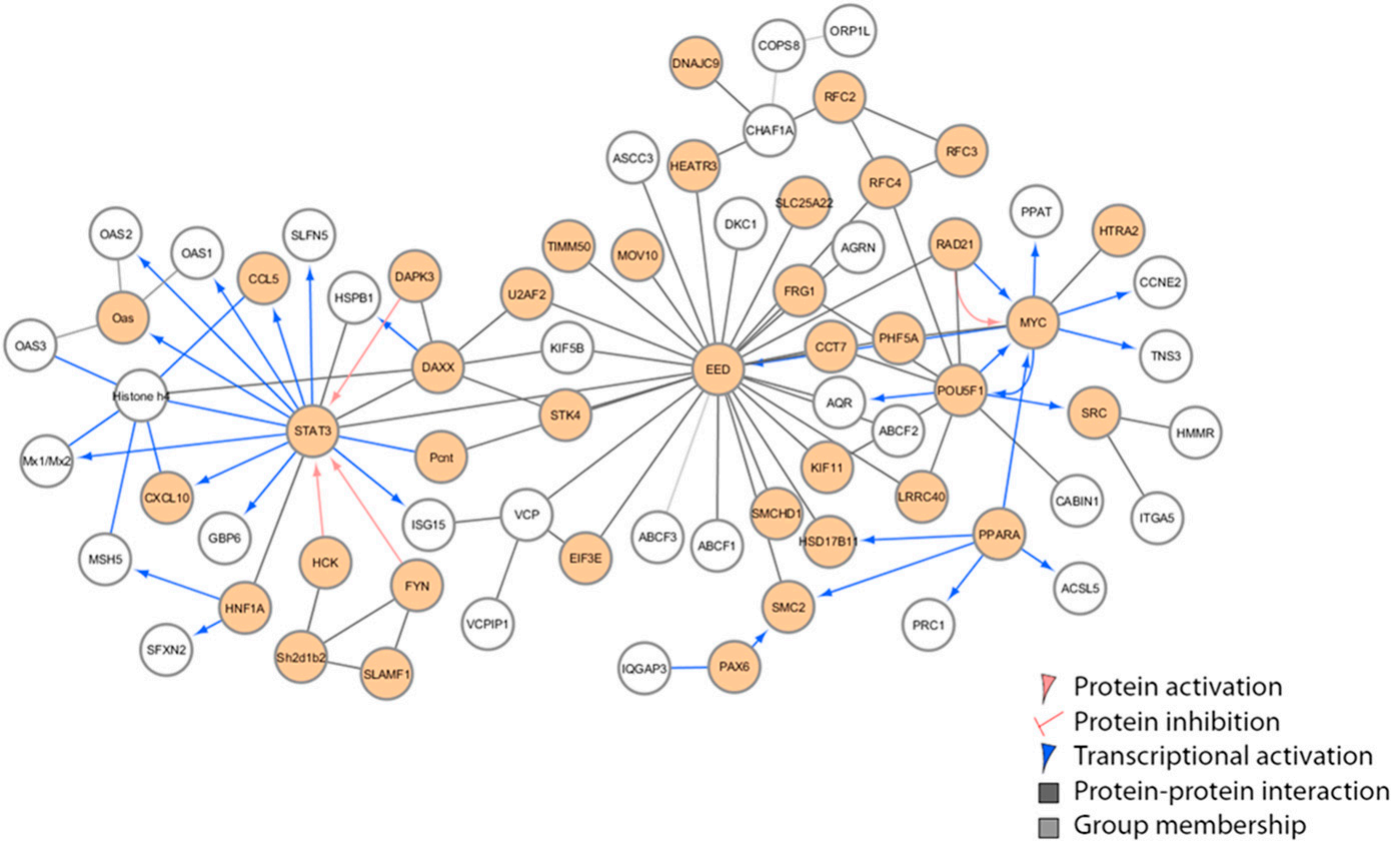
Innate immune regulation network [*Oas1b* allele (F/N)]. Innate immune regulatory network identified in F1s with F/N *Oas1b* alleles. Each node represents a gene with different colored lines showing types of connectivity. Filled-in nodes are target genes from the dataset.

**Figure 11:**
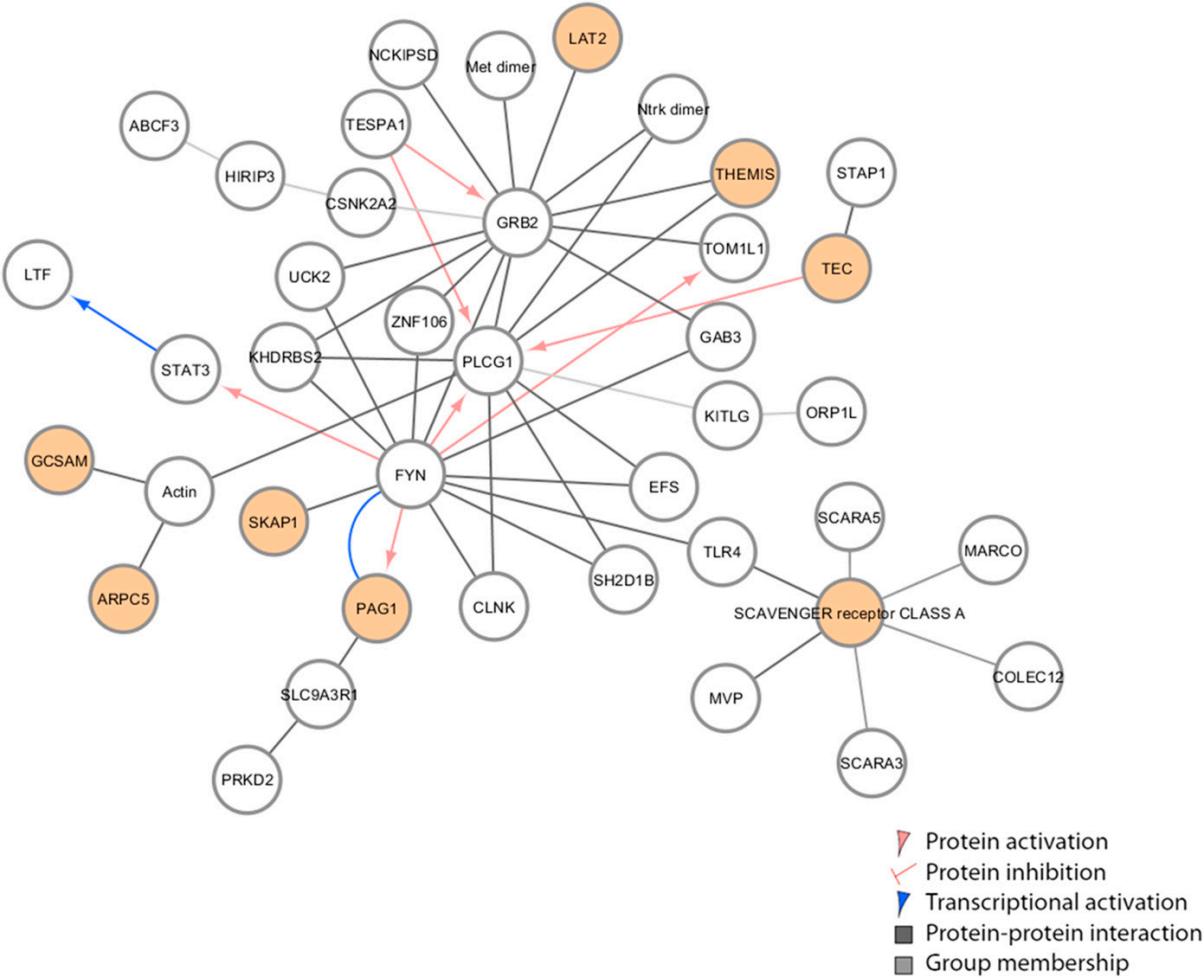
Innate immune regulation network [*Oas1b* allele (N/N)]. Innate immune regulatory network identified in F1s with N/N *Oas1b* alleles. Each node represents a gene with different colored lines showing types of connectivity. Filled-in nodes are target genes from the dataset.

Based on correlation and functional analysis, we proceeded to construct novel innate immune regulatory networks to discern the unique host response associated with *Oas1b* allelic differences. Using transcriptional data from F1s of different *Oas1b* haplotypes, we identified three immune regulatory networks during WNV infection. Figure 9 represents the *Oas1b* allele from both parents (F/F), Figure 10 shows the network from *Oas1b* heterozygous (F/N), and Figure 11 shows the network from *Oas1b* homozygous nonfunctioning (N/N) mice. The F/F innate (day 2 postinfection) immune network (Figure 9) is comprised of *Irf3*, *Irf7*, *Stat1*, *STAT2*, *Myc*, and the poly(ADP-ribose) polymerase family of proteins (PARP) response networks, and includes the *Oas* gene cluster. *Irf*s and *Stat*s were the major hubs launching virus-responsive genes and ISG networks, along with the induction of the *Oas* pathway. IRF3/7 also connects with several pattern recognition receptors including *DDX58* (the RLRs), *IFIH1*, and *DDX60*. PARP genes contribute to death receptor signaling and apoptosis while *Mx1/Mx2* are ISGs involved in antiviral innate immunity against several viruses (Shaffer *et al.* 2001). Antigen presentation is clearly activated, as indicated through network connections among the MHC class I family of genes. ABCF3 is connected by MYC (myelocytomatosis) and performs various roles in the cell, and has been identified as an *Oas1b*-binding protein of WNV control (Courtney *et al.* 2012). IFIT1B and *SNRPD3* (small nuclear ribonucleoprotein) connect to *ORP1L*, another *Oas1b*-binding protein. Thus, network analysis of F/F *Oas1b* mice reveals innate immune and antigen processing induction, OAS protein expression, and network interaction of known *Oas1b*-binding proteins in response to WNV associated with homozygous full-length *Oas1b* genotype.

The innate (day 2 postinfection) heterozygous network (F/N) in Figure 10 shared genetic signatures with F/F and included *STAT3* as one of the network hubs. Additional hubs included *EED* (embryonic ectoderm development), *MYC*, and *POU5F1* (POU class 5 homeo box 1), major cell signaling pathways (Neri *et al.* 2012; Shaw and Martin 2009). Along with *STAT3*, the heterozygous network also connects the OAS pathway genes (1–3 including *Oas1b*). Unique to this network is the incorporation of *DAXX* (death domain associated protein) and *Mov10* (RISC complex RNA helicase). *DAXX* is involved in death receptor signaling and targets infected cells for apoptosis (Engelhardt *et al.* 2001; Li *et al.* 2000; Perlman *et al.* 2001). *Mov10* is an IFN-inducible gene recently shown to have antiviral activity against RNA viruses like Sendai and VSV, but not seen before in WNV. This network again supports previous work (Courtney *et al.* 2012) where ABC proteins are binding partners to *Oas1b*, as noted above. In our network, we also identified connections with ABCF1 and ABCF2.

The N/N network, derived from later time points (days 7 and 12 postinfection), are involved in cell-to-cell signaling interactions and RNA post-translational modifications. There are several connector hubs in this network driven by genes *PLCG1*, GRB2, and *FYN*. *PLCG1* is involved in CD28 signaling in T helper cells and apoptosis. GRB2 is a growth factor receptor signaling-adaptor protein and is involved in a variety of cell signaling programs (Jang *et al.* 2009). Interestingly, ABCF3 is connected to GRPB2 through protein interactions with HIRIP3 (HIRA-interacting protein 3) and CSNK2A2 (casein kinase 2 α 2). It is believed that HIRIP3 functions as part of a multi-protein complex with roles in chromatin and histone metabolism. CSNK2A2 is a kinase, and has roles in apoptosis and G2/M phase cell cycling. ORP1L is connected to GRB2 through interactions with growth factor KITLG (Kit ligand). KITLG has several cellular roles including differentiation and apoptosis. The N/N network shares a STAT3 hub with the F/N network but its interaction partner is the FYN proto-oncogene (Src family tyrosine kinase family), which is involved in ATP binding as well as CD4 and CD8 receptor binding. Toll-like receptor 4 (TLR4), a well-documented immune signaling receptor, is downregulated. Taken together, these results link *Oas1b* haplotype with differential gene networks and WNV infection outcome.

### Identifying regulatory factors in host innate immune signaling

**Figure 12:**
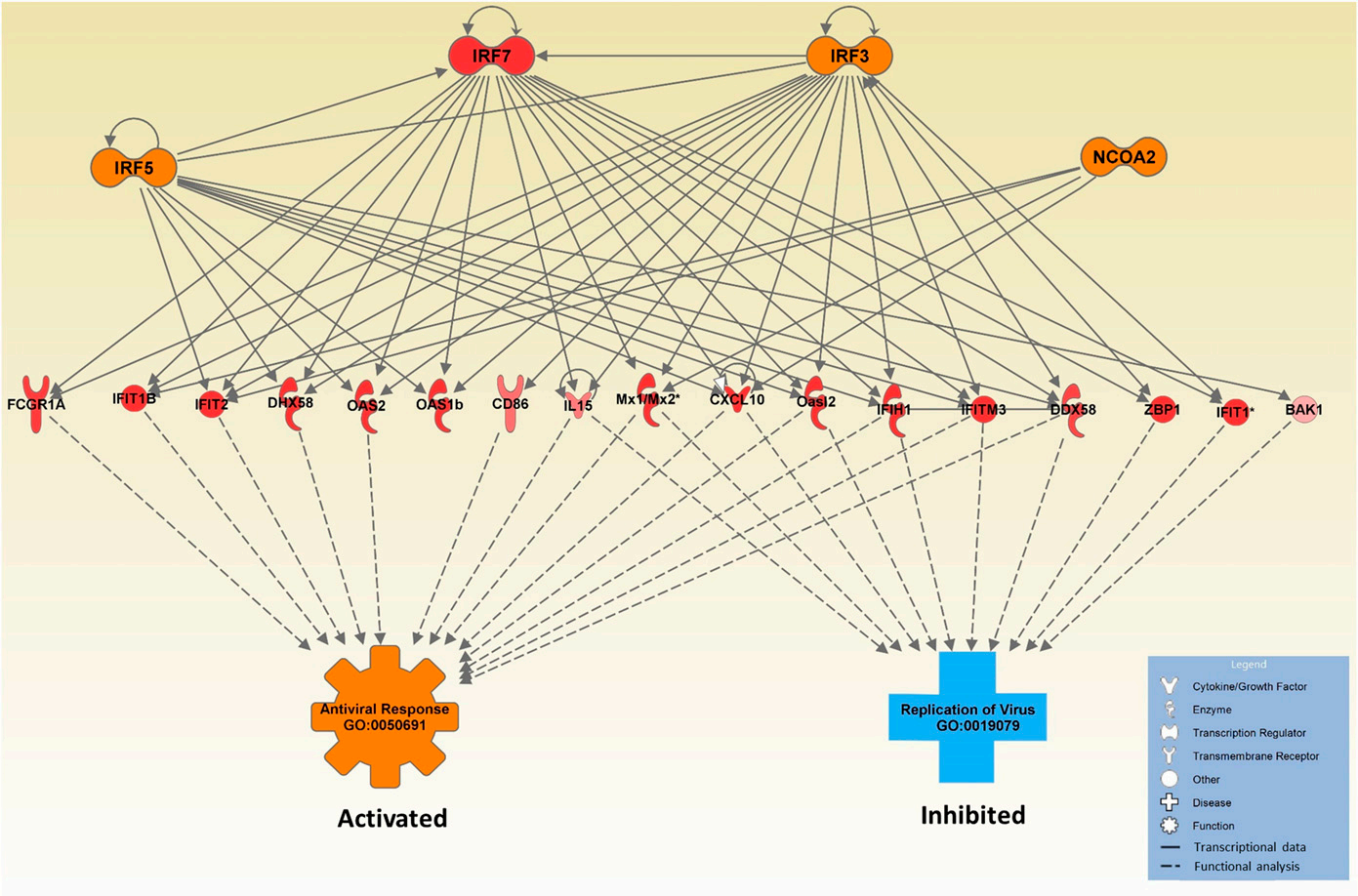
Infection regulator network F/F. A regulatory network generated from differentially expressed genes in CC(019x004)F1 at day 2 postinfection. The top nodes are transcriptional regulators. The red regulator was observed in the expression data and the orange nodes are predicted based on the target genes in our data set. The row of red-colored nodes indicate target genes that are upregulated. The lower orange and blue nodes refer to functional Gene Ontology (GO) terms. Antiviral response (GO:0050691) is predicted to be activated and replication of virus (GO:0019079) is predicted to be inhibited.

Our results show that F1s containing functional *Oas1b* alleles from both parents do not succumb to WNV infection. To determine the regulators of transcriptional responses in these *Oas1b* F/F F1s, we performed a predicted regulator analysis to localize target genes and find additional transcriptional regulators necessary for protection. Our regulator network (Figure 12) revealed that gene expression upregulation occurred predominantly in ISGs at day 2 postinfection. The network analysis determined activation of four transcriptional regulators (IRF3, IRF5, IRF7, and NCOA2). Three of these—IRF3, IRF5, and NCOA2—have been functionally linked to innate immune regulation or WNV infection in previous studies (Lazear *et al.* 2013; Flammer *et al.* 2010). The regulators and their candidate genes shared two distinct operations: antiviral response and inhibition of viral replication.

Among the target genes in the network were *Oas* members, *Mx1*, and various chemokines. Additionally, BAK1 (BCL antagonist/killer 1) and ZBP1 (Z-DNA binding protein 1) are present in the network and play roles in cell cycle regulation and proapoptotic activity; each are putative IRF-target genes. FCGR1A FC γ receptor is also linked to this network and is known to function in antigen presentation and removal of infected cells via apoptosis. NCOA2 is an *Oas1b* F/F network gene and is a transcriptional coregulatory protein with multiple nuclear interacting domains allowing DNA to become more accessible for transcription. *Oas1* and 2 expression is in part mediated by IRFs and NCOA2; studies using mouse bone-derived macrophages lacking NCOA2 expression showed a decrease in ISG expression (Flammer *et al.* 2010). Thus, WNV infection in the context of the F/F *Oas1b* haplotype engages specific IRF, IFN, and virus-responsive transcriptional networks that mediate protection from neuroinvasion and disease.

## Discussion

Infectious diseases cause a wide range of responses across genetically diverse populations. The use of genetically diverse MPP within the infectious disease community has been increasing with an effort to characterize (Iraqi *et al.* 2012; Ferris *et al.* 2013; Rasmussen *et al.* 2014; Graham *et al.* 2015, 2016) and genetically define the range of disease responses under host genetic control during viral infections (Boon *et al.* 2009; Ferris *et al.* 2013; Gralinski *et al.* 2015, 2017). Here, we confirmed a role for variants at the *Oas1b* locus in driving major disease outcomes following WNV infection. By leveraging prior gene sequence information of the *Oas1b* gene, we subclassified our F1s into four diplotype classes at the *Oas1b* locus. We found significant within-class variation in disease outcomes in these F1s, utilized transcriptional analysis to identify key host genes (and pathways) involved in allelic differences, and obtained a comprehensive view of the innate immune response associated with *Oas1b* haplotype variation.

### Genetic variation impacts disease

The *Oas1b* gene has been shown (Bigham *et al.* 2011; Courtney *et al.* 2012) to play a major role in controlling WNV disease by limiting viral replication (Kajaste-Rudnitski *et al.* 2006) within the C3H mouse strain (Perelygin *et al.* 2002). We identified a major-effect QTL over the *Oas1b* locus within a large population of F1s, and this QTL showed allele effects consistent with the previously described sequence variation within *Oas1b*. Importantly, this variant appears to segregate between wild-derived inbred strains and classical inbred strains derived from small population fancy mice (Yang *et al.* 2011). While our study and previous ones cannot definitively answer the question of whether the mutation arose in natural populations or in laboratory mice, it highlights the utility of assessing a broader range of genetic variants (both at targeted loci and genome-wide) in dissecting disease responses.

The presence of genes of major effect within genetic reference or other mapping populations can overshadow effects of other genetic variants cocirculating within these populations. As seen in our study and others (Ferris *et al.* 2013), it is possible to detect, transcriptionally characterize, and genetically map variants modifying disease responses in the presence of a major gene. As many pathogens and other diseases are impacted by large-effect genes (Ferris and Heise 2014), care in the design of experiments and their resultant analysis within the context of reference populations must be given. As we have shown here, studies further characterizing between-class responses at a molecular level can improve insight into disease mechanisms. Alternatively, studies such as F2 crosses or molecular investigations of strains within an allele class can identify specific variants modifying disease and will help to expand our knowledge of naturally polymorphic networks driving aberrant disease responses.

*Oas1b* has been shown to act independently from the RNase L pathway to mediate protection against WNV infection. OAS1b lacks OAS activity, and hence does not produce 2-5A that otherwise binds and activates RNaseL (Brinton and Perelygin 2003; Perelygin *et al.* 2002). In addition to ABC and ORP1L protein binding by OAS1b, our network analysis also observed additional factors implicated in *Oas* haplotype control of WNV infection including *DAXX* (mediated apoptosis), *Mov10* (antiviral), *GRB2* (cell signaling and leukocyte migration), and *Trafd1*. *Trafd1* has not previously been associated with the *Oas* family but appears within our QTL heatmap (Figure 5). *Trafd1* is an immune regulator known to be activated by STAT1 in mouse bone-derived macrophages (Mashima *et al.* 2005), and possibly contributes to innate immune protection mediated by this Oas1b network.

Evolutionary studies have observed that there is a balancing selection occurring at the *Oas1* gene locus across the human and nonhuman primate species. The allelic drift between primates produced variations in amino acids associated with RNA binding and this contributed to the susceptibility to flaviviruses (Ferguson *et al.* 2008). By performing a large genome scan across our diverse mouse backgrounds, we confirmed a QTL (containing the *Oas* genes and others) correlated with WNV infection. We chose here to observe the transcriptional differences influenced by *Oas1b* using the CC and allelic effects to determine the differential outcome (Figure S1). Our transcriptional analysis showed that there is still considerable strain-specific variation within the F1 backgrounds with functional *Oas1b*. While true disentangling of the relationship between genetic variants within these transcriptional networks and *Oas1b* allelic variants (*e.g.*, via genome editing *Oas1b* loci within CC strains) is beyond the scope of this study, our results strongly suggest that the anti-WNV effect of *Oas1b* leads to differential activation of molecular programs. Further work to understand these programs, the role of genetic variants within its members, and the causal role of *Oas1b*, will help to elucidate the mechanisms of WNV disease and point to potential therapeutic responses to this disease.

We observed for the first time the role of parental effects of *Oas1b* and their differential host transcriptomics. Among the heterozygous F1s, we noted a slightly higher survival rate in F1s where *Oas1b* was functional in the mother (F/N). We also observed three immune regulatory networks influenced by WNV infection and the *Oas1b* haplotype. Our analysis found a surprising relationship with cell cycle checkpoint signaling, diapedesis, and *Oas1b* haplotype. We found that the *Mov10* gene was included in this network of gene activation in F1s with heterozygous F/N alleles. Interestingly, *Mov10* has been shown previously to function in an IRF3-dependent manner independent of the RIG-I pathway (Cuevas *et al.* 2016), implicating possible RIG-I-independent signaling within the *Oas1b* network to drive gene expression within this module.

We were also able to assemble an innate immune network linking functional *Oas1b* to proteins including IRFs, specific ISGs, ubiquitin protein ligases, and PARP genes. The F/F and some F/N F1s displayed earlier viral recognition, which appeared in transcriptional pathway analysis and qPCR through *Ifit1* and *Ifn*β*1* expression. In symptomatic F1s lacking functional *Oas1b* (N/N), transcriptomics profiling indicated a lack of innate immune induction and cell migration that included monocyte migration and macrophage development. These observations indicate that, without activation of key processes such as innate immune signaling, diapedesis, and IFN responses, the host is unable to develop a strong defense and thus succumbs to infection in a manner linked to loss of *Oas1b* function. For example, the symptomatic N/F CC(055x028)F1 was unable to control viral replication in the spleen and developed neuroinvasive infection by day 12 (Figure S4). Lastly, our regulatory network (Figure 12) identified nuclear receptor coactivator 2 (NCOA2) as a novel transcriptional regulator producing an antiviral effect linked with *Oas1b*.

Understanding why some people are susceptible to viral infection while others are resistant is both a genetics and a genomics challenge that can be studied through genetics reference populations and infection models like the CC. Our study identifies new features of gene regulation linked with differential *Oas1b* genotypes within varied genetic backgrounds. This work also affirms the CC as a valuable tool for revealing the genetics of immune regulation and WNV control. CC mouse population is therefore an optimal model of virus infection amenable to defining genetic traits of disease outcome.

## Supplementary Material

Supplemental material is available online at http://www.g3journal.org/content/7/6/1665.supplemental.
