# Supplementary figures and images for: Oas1b-dependent Immune Transcriptional Profiles of West Nile Virus Infection in the Collaborative Cross
Source: G3 (Bethesda). 2017 Jun 5;7(6):1665–82. doi: 10.1534/g3.117.041624 (PMC5473748; doi:10.1534/g3.117.041624)

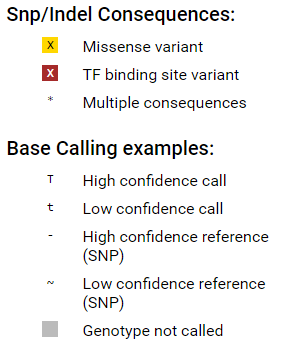

Supplement: Supplementary file 1 [file 1665FigureS1.key.tif]

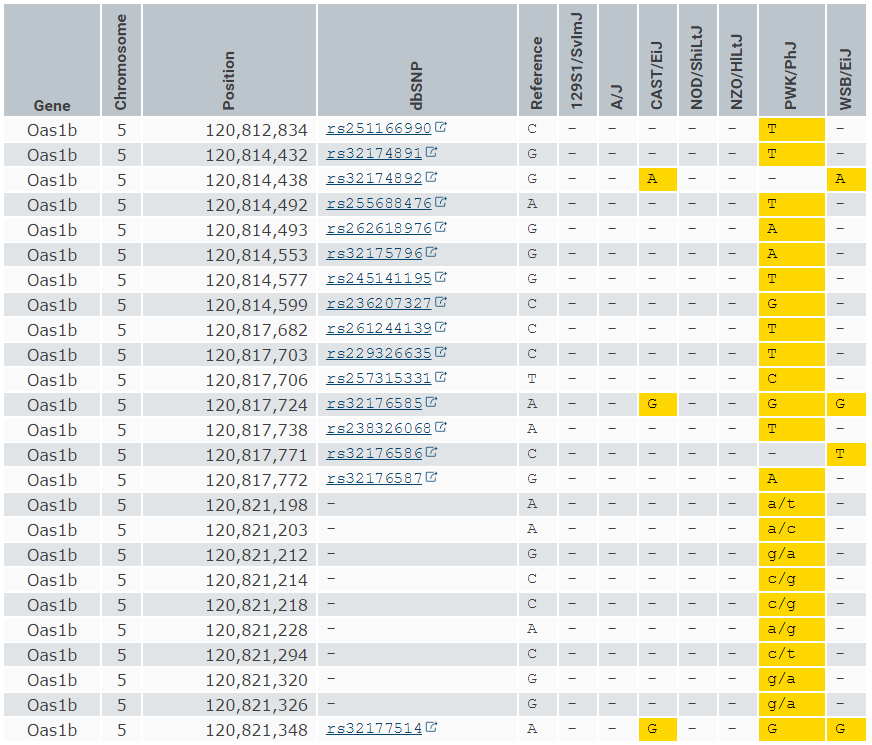

Supplement: Supplementary file 2 [file 1665FigureS1.tif]

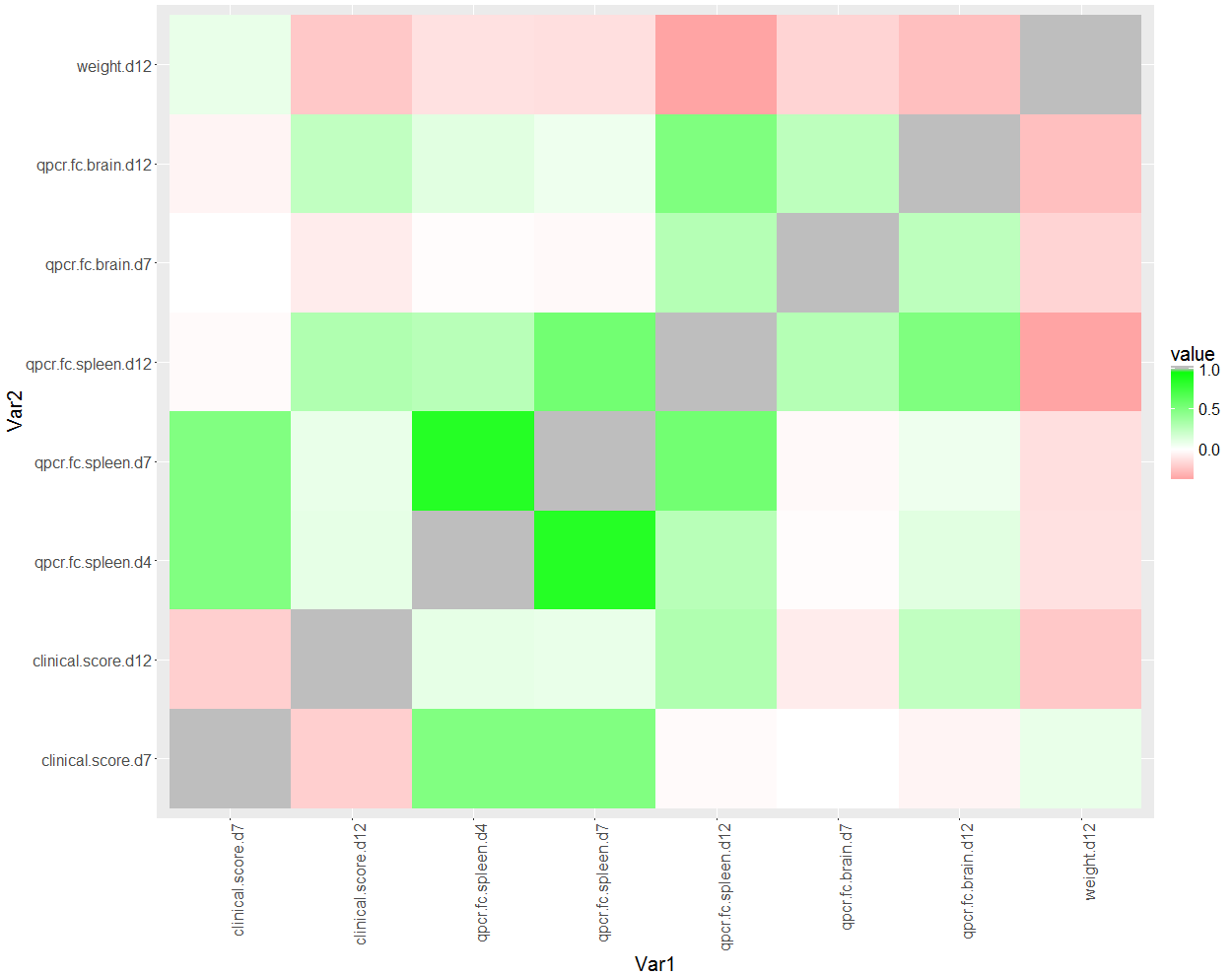

Supplement: Supplementary file 3 [file 1665FigureS2.tiff]

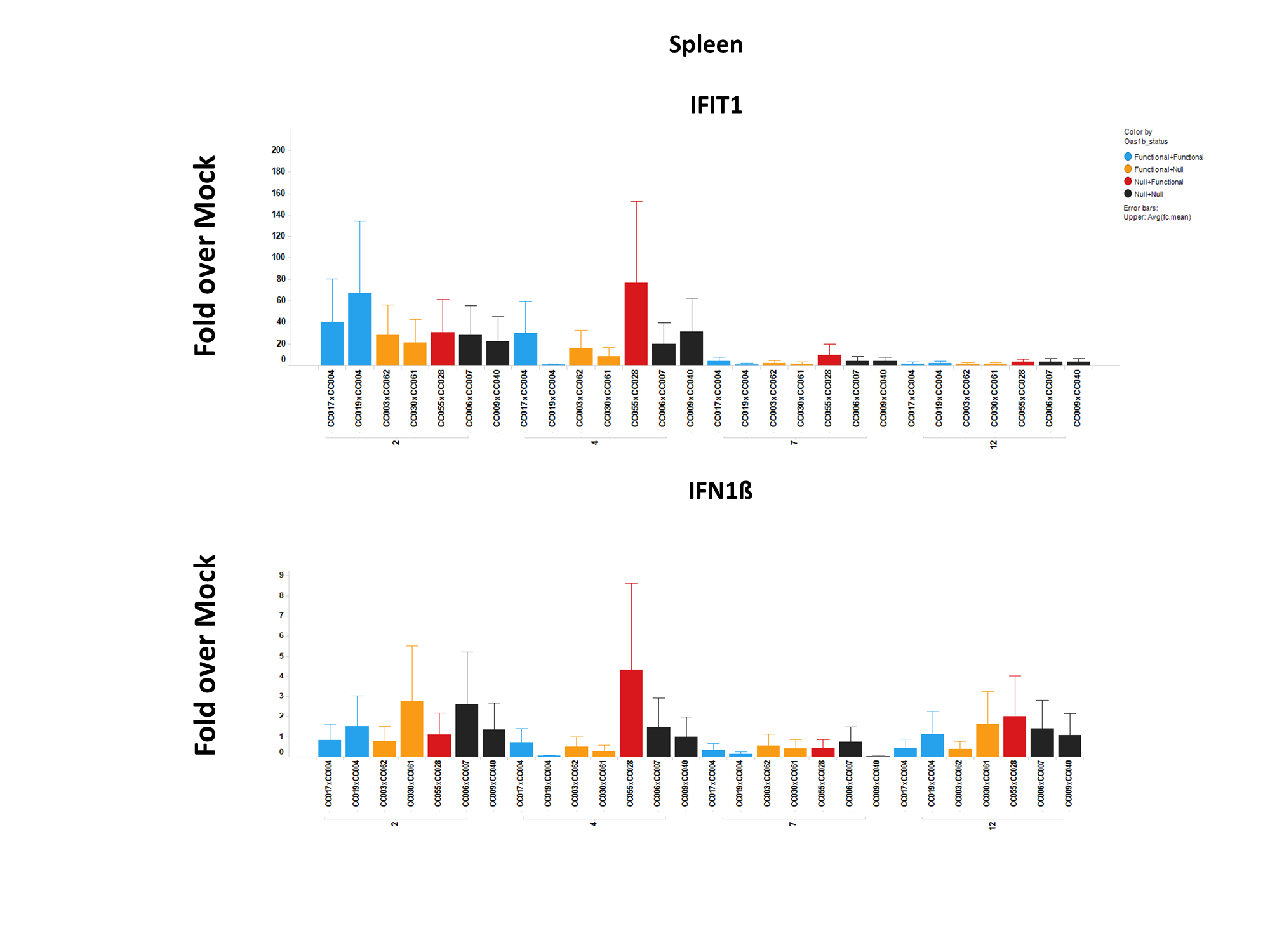

Supplement: Supplementary file 4 [file 1665FigureS3.tif]

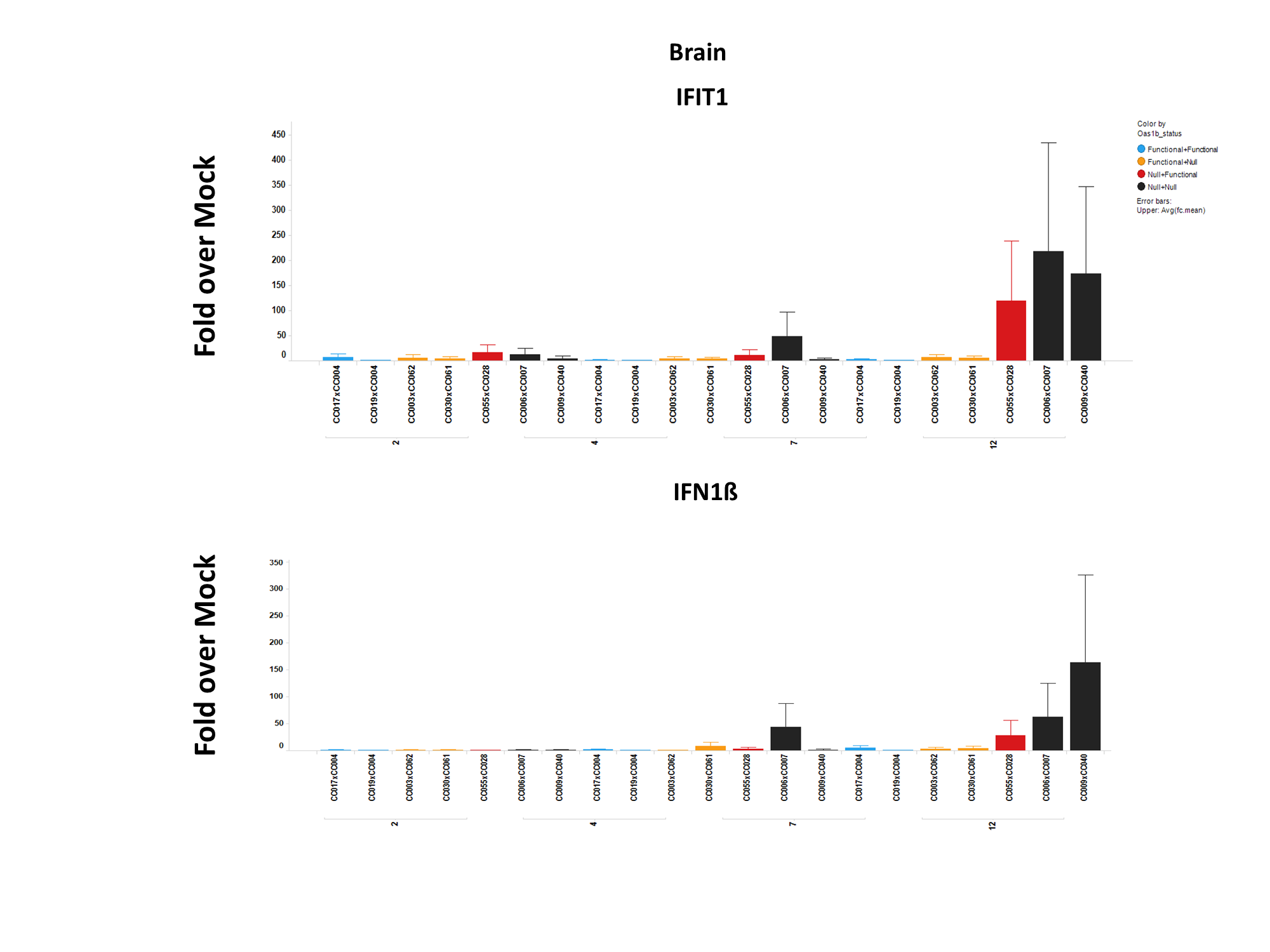

Supplement: Supplementary file 5 [file 1665FigureS4.tif]

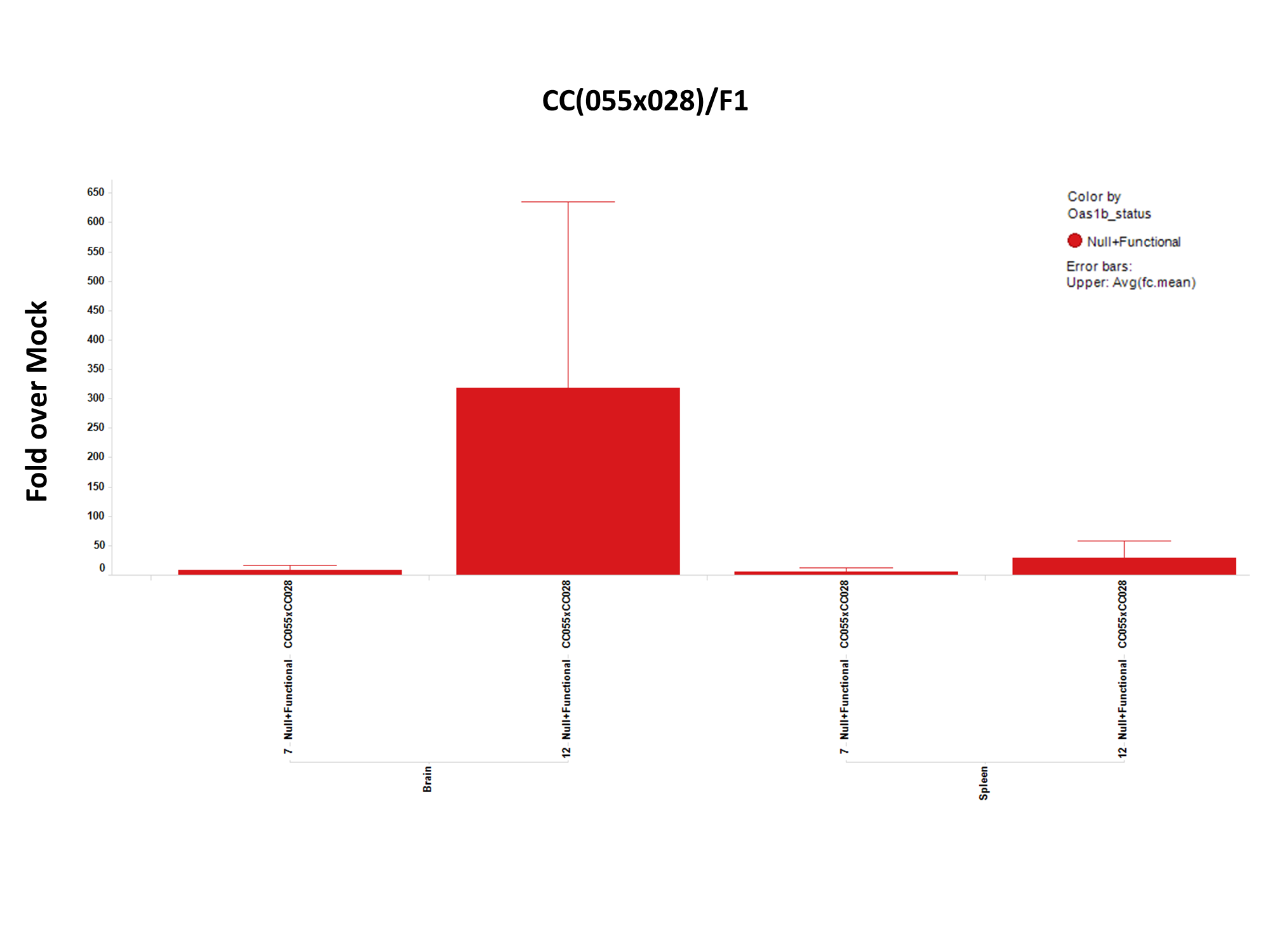

Supplement: Supplementary file 6 [file 1665FigureS5.tif]

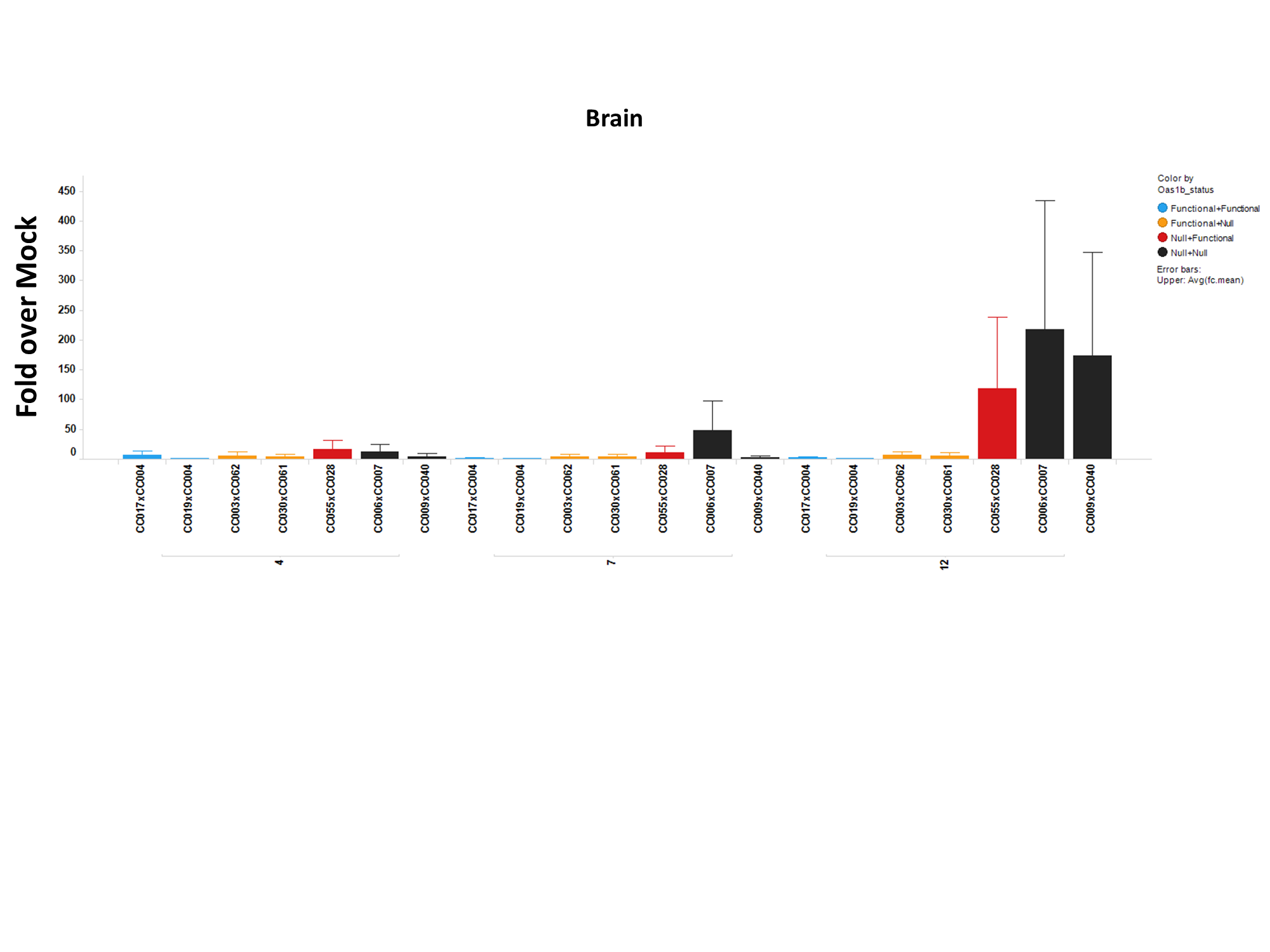

Supplement: Supplementary file 7 [file 1665FigureS6.tif]

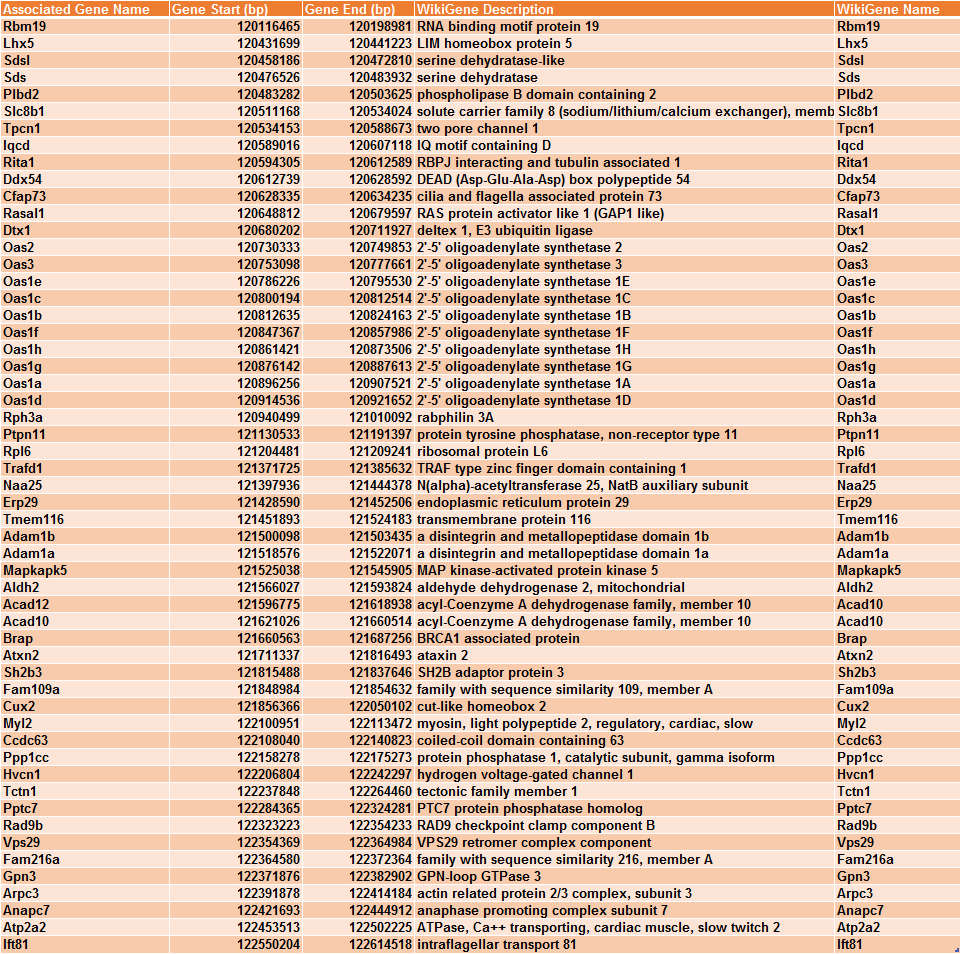

Supplement: Supplementary file 8 [file 1665TableS1.tif]

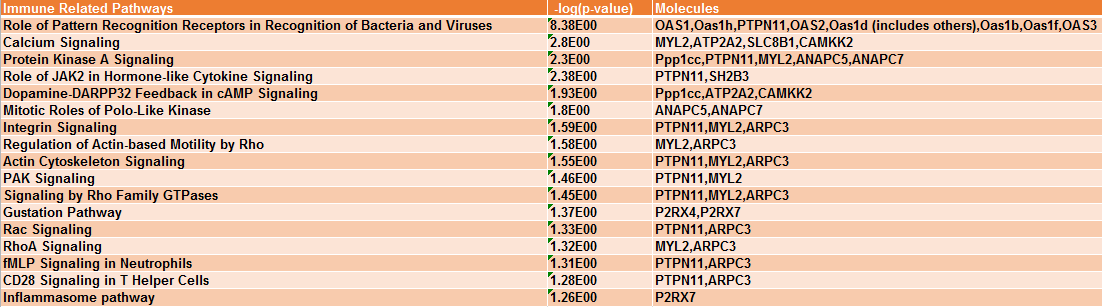

Supplement: Supplementary file 9 [file 1665TableS2.tif]
