# Supplementary material for: Oas1b-dependent Immune Transcriptional Profiles of West Nile Virus Infection in the Collaborative Cross
Source: G3 (Bethesda). 2017 Jun 5;7(6):1665–82. doi: 10.1534/g3.117.041624 (PMC5473748; doi:10.1534/g3.117.041624)
Supplement: Supplementary file 10 [file 1665CombinedSI.pdf]

Supplemental Figures

Gene	Chromosome	Position	dbSNP	Reference	129S1/SvImJ	A/J	CAST/EiJ	NOD/ShiLtJ	NZO/HILtJ	PWK/PhJ	WSB/EiJ
Oas1b	5	120,812,834	rs251166990	C	-	-	-	-	-	T	-
Oas1b	5	120,814,432	rs32174891	G	-	-	-	-	-	T	-
Oas1b	5	120,814,438	rs32174892	G	-	-	A	-	-	-	A
Oas1b	5	120,814,492	rs255688476	A	-	-	-	-	-	T	-
Oas1b	5	120,814,493	rs262618976	G	-	-	-	-	-	A	-
Oas1b	5	120,814,553	rs32175796	G	-	-	-	-	-	A	-
Oas1b	5	120,814,577	rs245141195	G	-	-	-	-	-	T	-
Oas1b	5	120,814,599	rs236207327	C	-	-	-	-	-	G	-
Oas1b	5	120,817,682	rs261244139	C	-	-	-	-	-	T	-
Oas1b	5	120,817,703	rs229326635	C	-	-	-	-	-	T	-
Oas1b	5	120,817,706	rs257315331	T	-	-	-	-	-	C	-
Oas1b	5	120,817,724	rs32176585	A	-	-	G	-	-	G	G
Oas1b	5	120,817,738	rs238326068	A	-	-	-	-	-	T	-
Oas1b	5	120,817,771	rs32176586	C	-	-	-	-	-	-	T
Oas1b	5	120,817,772	rs32176587	G	-	-	-	-	-	A	-
Oas1b	5	120,821,198	-	A	-	-	-	-	-	a/t	-
Oas1b	5	120,821,203	-	A	-	-	-	-	-	a/c	-
Oas1b	5	120,821,212	-	G	-	-	-	-	-	g/a	-
Oas1b	5	120,821,214	-	C	-	-	-	-	-	c/g	-
Oas1b	5	120,821,218	-	C	-	-	-	-	-	c/g	-
Oas1b	5	120,821,228	-	A	-	-	-	-	-	a/g	-
Oas1b	5	120,821,294	-	C	-	-	-	-	-	c/t	-
Oas1b	5	120,821,320	-	G	-	-	-	-	-	g/a	-
Oas1b	5	120,821,326	-	G	-	-	-	-	-	g/a	-
Oas1b	5	120,821,348	rs32177514	A	-	-	G	-	-	G	G

Snp/Indel Consequences:

- X Missense variant
- X TF binding site variant
- * Multiple consequences

Base Calling examples:

- T High confidence call
- t Low confidence call
- - High confidence reference (SNP)
- ~ Low confidence reference (SNP)
- Genotype not called

Figure S1. SNPs in Oas1b founders. A list of known single nucleotide polymorphisms found in the Oas1b region of chromosome 5. The table indicates which region had a variant and which of the eight founder strains contains that variant. Found with the Sanger SNP viewer: (https://www.sanger.ac.uk/sanger/Mouse_SnpViewer), (Keane et al., 2011),.

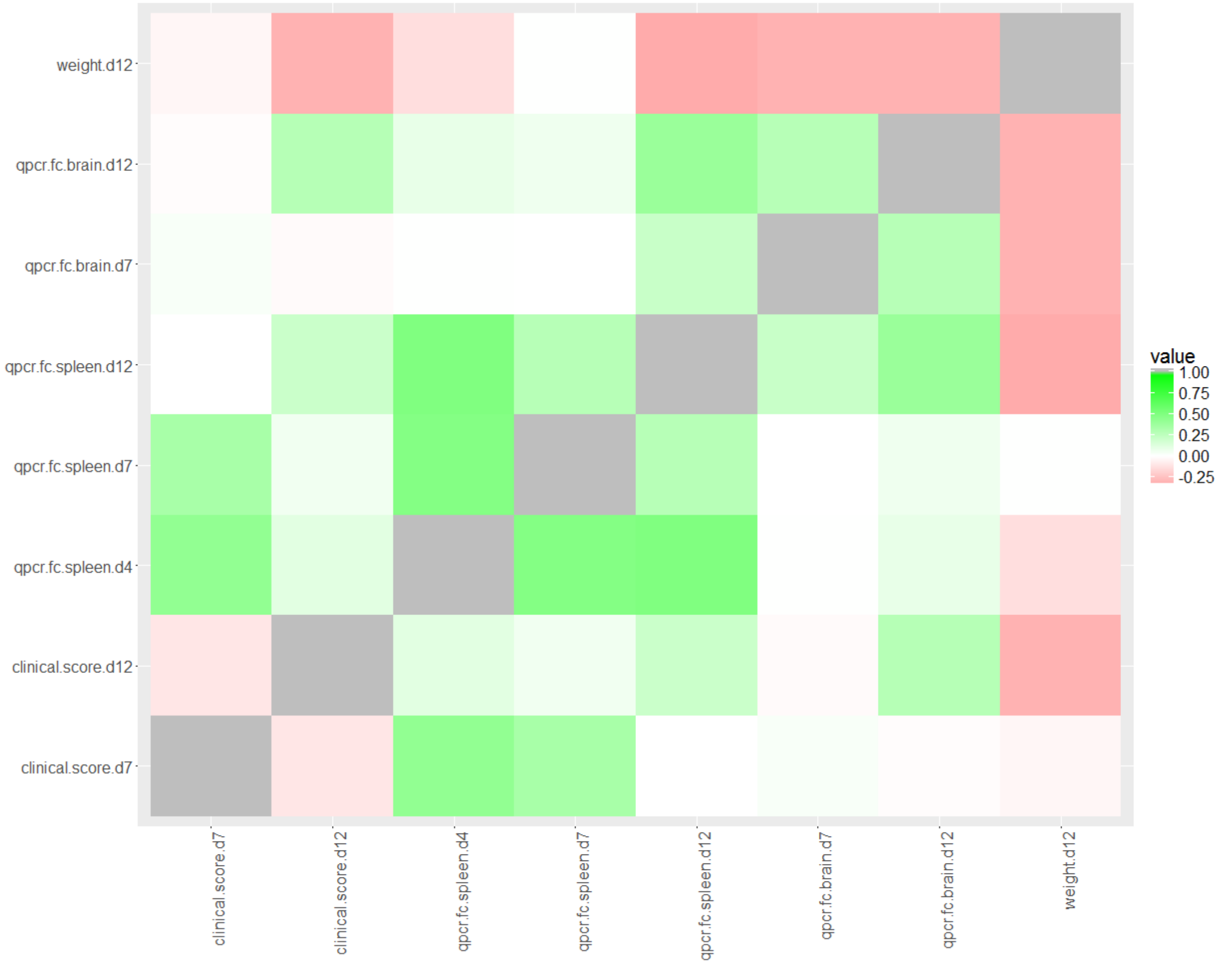

Figure S2. Correlation heat map of phenotypes

The heatmap shows the Pearson correlation scores representing relationships between phenotypes (clinical score, qPCR, and weight loss at day 12 post infection), with phenotypes represented on the X and Y axes. The green scale represents the value of positive correlation between phenotypes. The red scale represents negative correlation.

Spleen

IFIT1

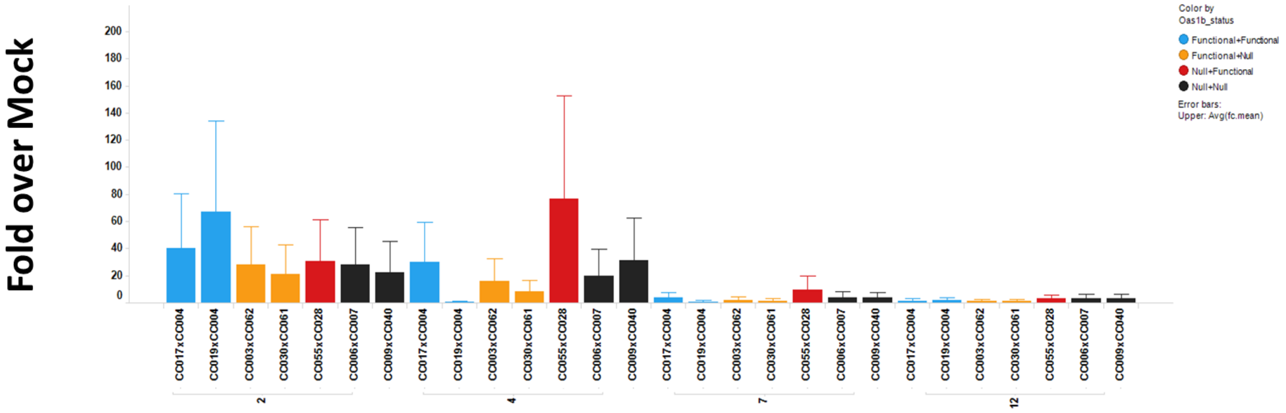

IFN1ß

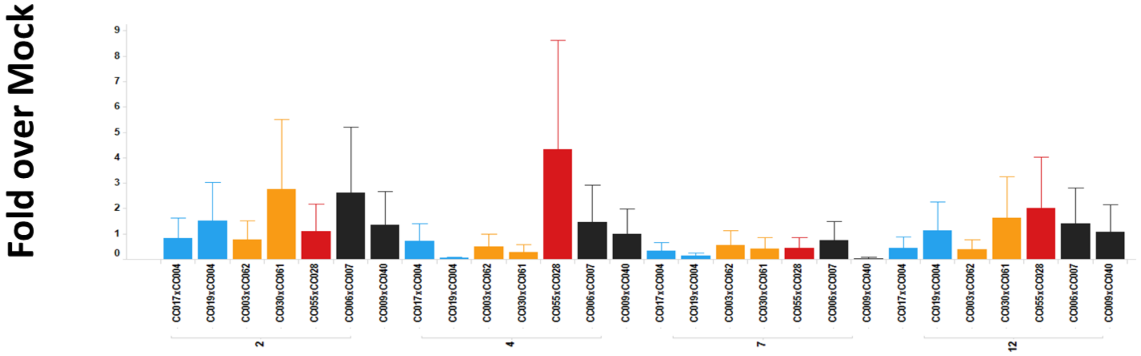

Figure S3. qPCR for IFIT1 and IFNb1 in the spleen

The y-axis shows fold change over mock averaged over each RIX background and the x-axis shows CC line IDs followed by days' post infection (2, 4 ,7). Each color represents a different Oas1b designation. Blue=F/F, Orange=F/N, Red=N/F, Black=N/N.

Brain

IFIT1

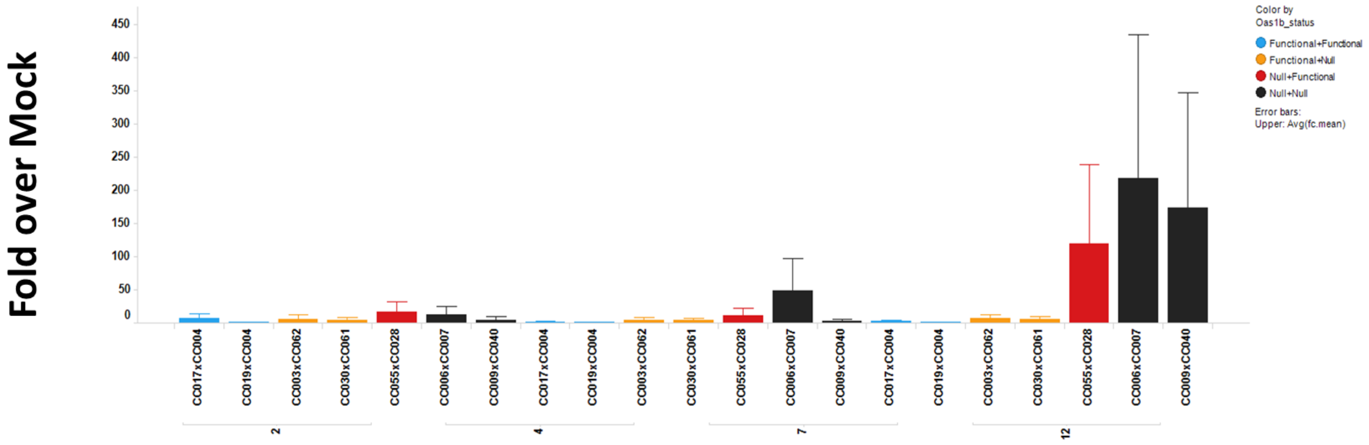

IFN1ß

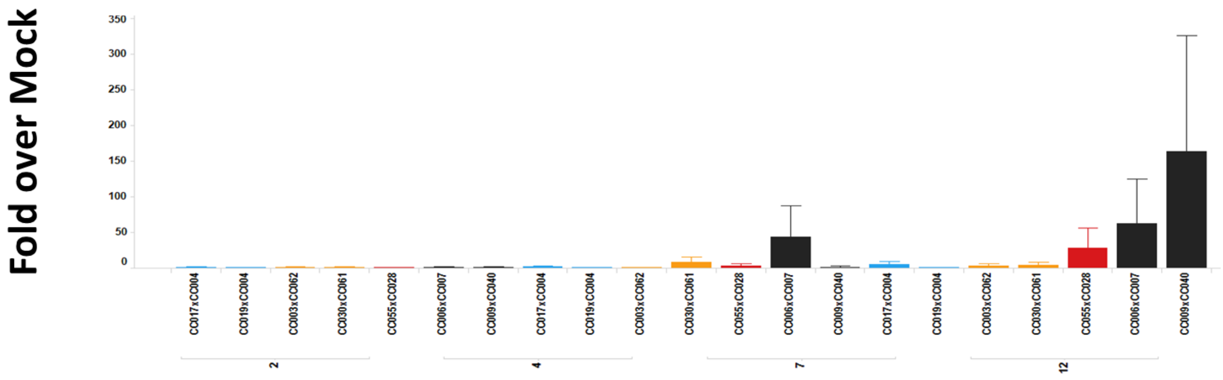

Figure S4. qPCR for IFIT1 and IFNb1 in the brain

The y-axis shows fold change over mock in the RIX lines selected for transcriptomics. The x-axis shows CC line IDs followed by days' post infection (2, ,4 ,7). Each color represents a different Oas1b designation. Blue=F/F, Orange=F/N, Red=N/F, Black=N/N.

CC(055x028)/F1

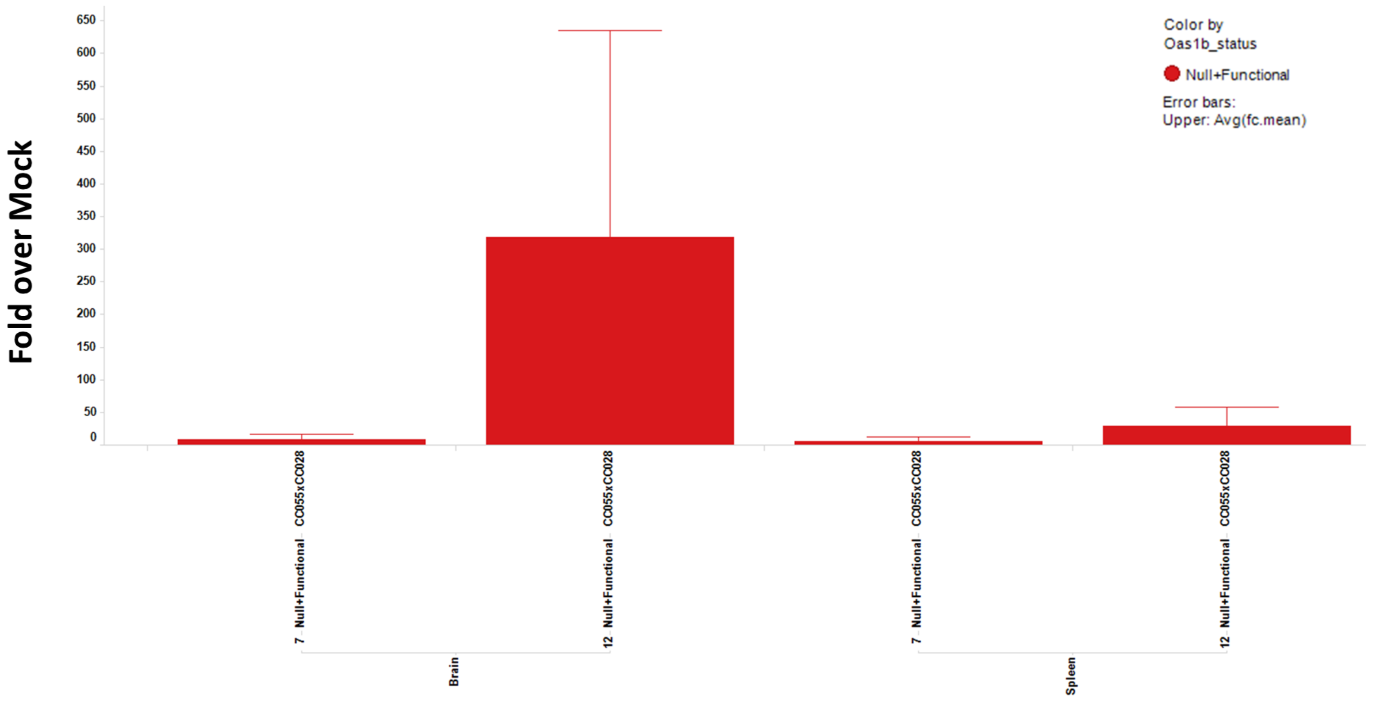

Figure S5. qPCR of WNV in CC(055x028)/F1 at days' 7 and 12 in the brain and spleen.

The y-axis shows fold change over mock for RIX CC(055x028)/F1. The x-axis shows days' post infection (2, ,4 ,7). The top part of the figure is spleen and the lower part is brain. The color here represents its haplotype (Red=N/F).

Brain

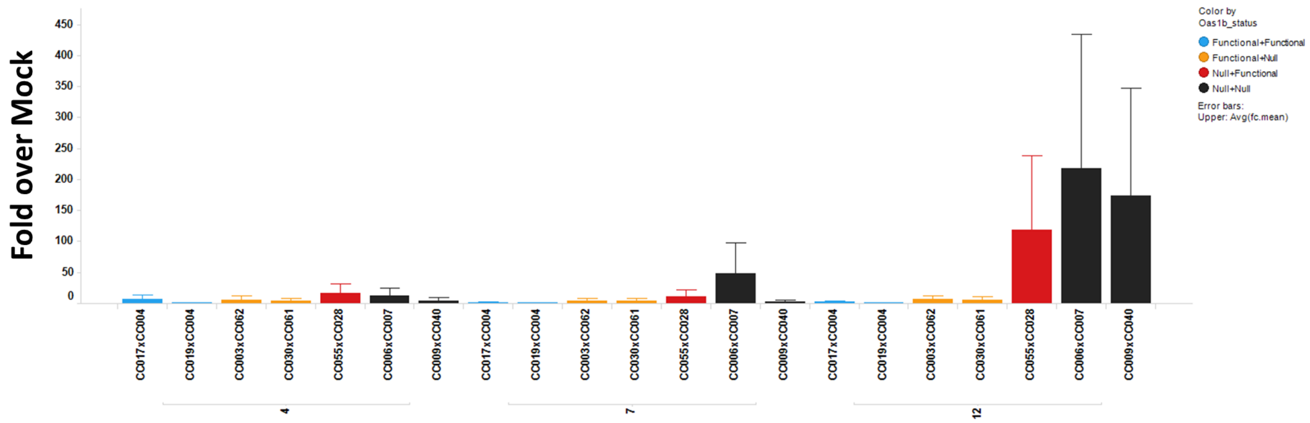

Figure S6. qPCR of WNV in the brain of selected CC RIX lines at day 4, 7, and 12 post infection

The y-axis shows fold change over mock in the RIX lines selected for transcriptomics. The x-axis shows days' post infection (4 ,7, 12). Each color represents a different Oas1b designation. Blue=F/F, Orange=F/N, Red=N/F, Black=N/N.

Associated Gene Name	Gene Start (bp)	Gene End (bp)	WikiGene Description	WikiGene Name
Rbm19	120116465	120198981	RNA binding motif protein 19	Rbm19
Lhx5	120431699	120441223	LIM homeobox protein 5	Lhx5
Sdsl	120458186	120472810	serine dehydratase-like	Sdsl
Sds	120476526	120483932	serine dehydratase	Sds
Plbd2	120483282	120503625	phospholipase B domain containing 2	Plbd2
Slc8b1	120511168	120534024	solute carrier family 8 (sodium/lithium/calcium exchanger), memb	Slc8b1
Tpcn1	120534153	120588673	two pore channel 1	Tpcn1
Iqcd	120589016	120607118	IQ motif containing D	Iqcd
Rita1	120594305	120612589	RBPJ interacting and tubulin associated 1	Rita1
Ddx54	120612739	120628592	DEAD (Asp-Glu-Ala-Asp) box polypeptide 54	Ddx54
Cfap73	120628335	120634235	cilia and flagella associated protein 73	Cfap73
Rasal1	120648812	120679597	RAS protein activator like 1 (GAP1 like)	Rasal1
Dtx1	120680202	120711927	deltex 1, E3 ubiquitin ligase	Dtx1
Oas2	120730333	120749853	2'-5' oligoadenylate synthetase 2	Oas2
Oas3	120753098	120777661	2'-5' oligoadenylate synthetase 3	Oas3
Oas1e	120786226	120795530	2'-5' oligoadenylate synthetase 1E	Oas1e
Oas1c	120800194	120812514	2'-5' oligoadenylate synthetase 1C	Oas1c
Oas1b	120812635	120824163	2'-5' oligoadenylate synthetase 1B	Oas1b
Oas1f	120847367	120857986	2'-5' oligoadenylate synthetase 1F	Oas1f
Oas1h	120861421	120873506	2'-5' oligoadenylate synthetase 1H	Oas1h
Oas1g	120876142	120887613	2'-5' oligoadenylate synthetase 1G	Oas1g
Oas1a	120896256	120907521	2'-5' oligoadenylate synthetase 1A	Oas1a
Oas1d	120914536	120921652	2'-5' oligoadenylate synthetase 1D	Oas1d
Rph3a	120940499	121010092	rabphilin 3A	Rph3a
Ptpn11	121130533	121191397	protein tyrosine phosphatase, non-receptor type 11	Ptpn11
Rpl6	121204481	121209241	ribosomal protein L6	Rpl6
Trafd1	121371725	121385632	TRAF type zinc finger domain containing 1	Trafd1
Naa25	121397936	121444378	N(alpha)-acetyltransferase 25, NatB auxiliary subunit	Naa25
Erp29	121428590	121452506	endoplasmic reticulum protein 29	Erp29
Tmem116	121451893	121524183	transmembrane protein 116	Tmem116
Adam1b	121500098	121503435	a disintegrin and metallopeptidase domain 1b	Adam1b
Adam1a	121518576	121522071	a disintegrin and metallopeptidase domain 1a	Adam1a
Mapkapk5	121525038	121545905	MAP kinase-activated protein kinase 5	Mapkapk5
Aldh2	121566027	121593824	aldehyde dehydrogenase 2, mitochondrial	Aldh2
Acad12	121596775	121618938	acyl-Coenzyme A dehydrogenase family, member 10	Acad10
Acad10	121621026	121660514	acyl-Coenzyme A dehydrogenase family, member 10	Acad10
Brap	121660563	121687256	BRCA1 associated protein	Brap
Atxn2	121711337	121816493	ataxin 2	Atxn2
Sh2b3	121815488	121837646	SH2B adaptor protein 3	Sh2b3
Fam109a	121848984	121854632	family with sequence similarity 109, member A	Fam109a
Cux2	121856366	122050102	cut-like homeobox 2	Cux2
Myl2	122100951	122113472	myosin, light polypeptide 2, regulatory, cardiac, slow	Myl2
Ccdc63	122108040	122140823	coiled-coil domain containing 63	Ccdc63
Ppp1cc	122158278	122175273	protein phosphatase 1, catalytic subunit, gamma isoform	Ppp1cc
Hvcn1	122206804	122242297	hydrogen voltage-gated channel 1	Hvcn1
Tctn1	122237848	122264460	tectonic family member 1	Tctn1
Pptc7	122284365	122324281	PTC7 protein phosphatase homolog	Pptc7
Rad9b	122323223	122354233	RAD9 checkpoint clamp component B	Rad9b
Vps29	122354369	122364984	VPS29 retromer complex component	Vps29
Fam216a	122364580	122372364	family with sequence similarity 216, member A	Fam216a
Gpn3	122371876	122382902	GPN-loop GTPase 3	Gpn3
Arpc3	122391878	122414184	actin related protein 2/3 complex, subunit 3	Arpc3
Anapc7	122421693	122444912	anaphase promoting complex subunit 7	Anapc7
Atp2a2	122453513	122502225	ATPase, Ca++ transporting, cardiac muscle, slow twitch 2	Atp2a2
Ift81	122550204	122614518	intraflagellar transport 81	Ift81

Table S1. QTL gene table

A list of known genes identified in our day 12 weight loss QTL (2.5 megabases). This list excluded predicted genes.

Immune Related Pathways	-log(p-value)	Molecules
Role of Pattern Recognition Receptors in Recognition of Bacteria and Viruses	8.38E00	OAS1,Oas1h,PTPN11,OAS2,Oas1d (includes others),Oas1b,Oas1f,OAS3
Calcium Signaling	2.8E00	MYL2,ATP2A2,SLC8B1,CAMKK2
Protein Kinase A Signaling	2.3E00	Ppp1cc,PTPN11,MYL2,ANAPC5,ANAPC7
Role of JAK2 in Hormone-like Cytokine Signaling	2.38E00	PTPN11,SH2B3
Dopamine-DARPP32 Feedback in cAMP Signaling	1.93E00	Ppp1cc,ATP2A2,CAMKK2
Mitotic Roles of Polo-Like Kinase	1.8E00	ANAPC5,ANAPC7
Integrin Signaling	1.59E00	PTPN11,MYL2,ARPC3
Regulation of Actin-based Motility by Rho	1.58E00	MYL2,ARPC3
Actin Cytoskeleton Signaling	1.55E00	PTPN11,MYL2,ARPC3
PAK Signaling	1.46E00	PTPN11,MYL2
Signaling by Rho Family GTPases	1.45E00	PTPN11,MYL2,ARPC3
Gustation Pathway	1.37E00	P2RX4,P2RX7
Rac Signaling	1.33E00	PTPN11,ARPC3
RhoA Signaling	1.32E00	MYL2,ARPC3
fMLP Signaling in Neutrophils	1.31E00	PTPN11,ARPC3
CD28 Signaling in T Helper Cells	1.28E00	PTPN11,ARPC3
Inflammasome pathway	1.26E00	P2RX7

Table S2. Table of pathways found enriched in QTL (4 megabases)

A list of known pathways within the large QTL identified with other phenotypes (see table 1).
