# Supplementary material for: Oas1b-dependent Immune Transcriptional Profiles of West Nile Virus Infection in the Collaborative Cross
Source: G3 (Bethesda). 2017 Jun 5;7(6):1665–82. doi: 10.1534/g3.117.041624 (PMC5473748; doi:10.1534/g3.117.041624)
Supplement: Supplementary file 12 [file 1665Green_Oas1b_data_policy_documentation.docx]

Oas1b-dependent Immune Transcriptional Profiles of West Nile Virus Infection in the Collaborative Cross

**Richard Green**, Courtney Wilkins, Sunil Thomas, Aimee Sekine, Duncan M. Hendrick, Kathleen Voss, Renee C. Ireton, Michael Mooney, Jennifer T. Go, Gabrielle Choonoo, Sophia Jeng, Fernando Pardo-Manuel de Villena, Martin T. Ferris, Shannon McWeeney, and Michael Gale, Jr.

1. Genetic material.
2. For a complete list of CC genomes and line information please refer to the following:
3. <http://csbio.unc.edu/CCstatus/index.py?run=Pseudo>
4. CC lines used in QTL analysis:
5. <https://github.com/greener98103/oas1b/blob/master/cc_lines_screened_qtl.txt>
6. CC lines used in qPCR: <https://github.com/greener98103/oas1b/blob/master/cc_lines_screened_qPCR.txt>
7. CC lines used in determining outcome: <https://github.com/greener98103/oas1b/blob/master/cc_lines_screened_outcome.txt>
8. Additional information: CC lines are available for purchase from the UNC Systems Genetics Core at (<http://csbio.unc.edu/CCstatus/index.py>). This material has been referenced in previous articles: (<http://csbio.unc.edu/CCstatus/index.py?run=CCStatusStats.genetics>, <https://www.ncbi.nlm.nih.gov/m/pubmed/22345608/>)
9. Raw Genotypes.
10. Genotypes were generated on the MUGA mouse array and extracted using Illumina BeadStudo software. This data is available from the Mutant Mouse Regional Resource Center at UNC’s website (https://www.med.unc.edu/mmrrc/genotypes/publications). The data includes raw x- and y- intensities, and derived genotype calls.
11. Variants files were extracted from recent Variant Calling Files VCFs using Sanger's recent sequencing methods. The version build 38 used and can be found here: (<ftp://ftp-mouse.sanger.ac.uk/REL-1303-SNPs_Indels-GRCm38/>
12. Information on Sangers re-sequencing methods can be found here:(<http://www.sanger.ac.uk/science/data/mouse-genomes-project>)
13. Additional marker information can be found here:(<http://csbio.unc.edu/CCstatus/index.py?run=MugaPlatform>)
14. To determine Oas1b status, founder genotype probabilities were obtained for the marker closest to the Oas1b locus ('UNC10037634', 12kb upstream of gene). Probabilities at this marker were then summed for different allele groups: 1) the 5 classical inbred strains (Null), and 2) WSB, Cast, PWK (Functional). The second group can be split into two (WSB/Cast vs. PWK) (see manuscript results for more information). The complete list can be found here:(<https://github.com/greener98103/oas1b/blob/master/oas1b_dam_sire_v2_status.csv>)
15. Raw Phenotype Data.
16. Supplemental file S1 contains the raw phenotypic data used in the QTL analysis (D12 weight percentage in 90 CC RIXes), as well as UNC CC IDs. This file is in csv format. This file can also be found on the Github page:(<https://github.com/greener98103/oas1b/blob/master/d12_weight_pheno_table_GaleOnly_90lines_Oas1b.txt>). This phenotype file also includes the Oas1b status of each animal. The following columns are included: dam_null_prob = probability of Null allele from dam dam_p_prob = probability of PWK allele from dam dam_wc_prob = probability of WSB or Cast allele from dam (same 3 columns as above for sire) Oas1b_status = the categories that we use when performing Oas1b-adjusted mapping analyses ("High" = at least one WSB/Cast allele, "Mod" = Null/PWK or PWK/Null, "Low" = Null/Null; Note: there are no PWK/PWK homozygotes)

4. Description of Phenotypes.

a. Weight loss – D12 weight change percentage (relative to D0) from CC mice infected with West Nile Virus. Link to data: (https://github.com/greener98103/oas1b/blob/master/d12_weight_pheno_table_GaleOnly_90lines_Oas1b.txt)

b. qPCR – Using SYBR Green (Applied Biosystems) RT-PCR, WNV was quantified relative to GAPDH by probing cDNA with WNV-specific probes in spleen and brain tissue. qPCR results were recorded as fold change over mock infected mice as described in the materials and methods. Link to data: (https://github.com/greener98103/oas1b/blob/master/qPCR_and_outcomes.csv)

c. Clinical Scores – The clinical scoring system used to evaluate WNV-infected mice was as follows: 0, healthy mouse (baseline); 1, ruffled fur, lethargy, hunched posture, no paresis, normal gait; 2, altered gait, limited movement in one hind limb; 3, lack of movement, paresis in one or both hind limbs; 4, moribund. Link to data: (https://github.com/greener98103/oas1b/blob/master/oas1b_clinical_scores.csv)

d. Outcome – To quantify disease outcome, we used weight loss and clinical scoring to segregate the CC RIX lines into two broad pathogenic phenotype categories: asymptomatic or symptomatic. Three animals were assessed for each CC RIX line and outcome was based on at least one mouse out of three meeting weight loss or clinical score criteria at any time point. Symptomatic were defined as having weight loss greater than 10% of original pre-infection weight, clinical score > 1, and/or death, whereas asymptomatic was defined as having weight loss < 10% of original pre-infection weight, clinical score of 0 or 1, and no death. Link to data: (<https://github.com/greener98103/oas1b/blob/master/qPCR_and_outcomes.csv>)

1. Filtered genotype file.
   1. Please see above, in the MMRRC download.
2. Filtered and normalized Phenotype file.
   1. Both raw and normalized expression data can be found in GEO, accession number: GSE91003.

1. Results file.
2. The results for the QTL scan for D12 weight change percentage (relative to D0), for all autosomal markers are in the link below. This includes all values from the DOQTL package returns, most relevant are columns: marker, chromosome, position.B38_Mb, lod, p ,and neg.log10.p (<https://raw.githubusercontent.com/greener98103/oas1b/master/results.csv>). An adjusted p-value for each marker was not included because permutation test was used to determine statistical significance of the LOD scores. The LOD score threshold for significance at p=0.05 is 6.75, and for p=0.1 is 6.31. The significant peak on chromosome 5 has a LOD of 7.96 and the Bayes Credible Interval is: Chr5: 119.9Mb-122.9Mb
3. Results from the transcriptional analysis are in the link below. These results include both the un-adjusted and adjusted p-values. Link to Data:( <https://github.com/greener98103/oas1b/wiki/Transcriptional-analysis>)
4. The QTL peak identified can be found here: <https://github.com/greener98103/oas1b/blob/master/d12_weight_Gale_chrom5_qtl.pdf>
5. Simulation studies.
6. No simulations were conducted.
7. Software
8. Information about the QTL workflow can be found here: <https://github.com/biodev/SIG/blob/master/SIG_QTL_Mapping_Workflow.ipynb>
9. Executable R code for QTL analysis: <https://github.com/greener98103/oas1b/blob/master/SIG_QTL_Mapping_Workflow_D12_Weight_Gale.r>
10. QTL mapping was performed using the DOQTL package which is publicly available through the CRAN depository as a standard R install.
11. Differential expression was performed using the Limma which is publicly available through the CRAN depository as a standard R install. More information on linear modeling analysis in R using microarray data can be found here:(http://www.bioconductor.org/packages/devel/bioc/vignettes/limma/inst/doc/usersguide.pdf)
12. Correlation analysis was performed using cor function in R which is publicly available and part of the base functions in the R-programming language.
13. Spotfire (http://spotfire.tibco.com, version 7.5.0.86) to produce box plots and graphs.
